# High-resolution promoter interaction analysis implicates genes involved in activation of type 3 innate lymphoid cells in immune disease risk

**DOI:** 10.1038/s41588-026-02681-0

**Published:** 2026-08-04

**Authors:** Valeriya Malysheva, Helen Ray-Jones, Nora Lakes, Rachel A. Brown, Tareian A. Cazares, Owen Clay, David E. Ohayon, Pavel Artemov, Joseph A. Wayman, Zi F. Yang, Monica Della Rosa, Carmen Petitjean, Clarissa Booth, Joseph I. J. Ellaway, Jenna R. Barnes, Andrew W. Dangel, Ankita Saini, William R. Orchard, Xiaoting Chen, Sreeja Parameswaran, Frances Burden, Mattia Frontini, Takashi Nagano, Peter Fraser, Stefan Schoenfelder, Matthew T. Weirauch, Leah C. Kottyan, David F. Smith, Nick Powell, Jill M. Weimer, Eugene M. Oltz, Chris Wallace, Emily R. Miraldi, Stephen N. Waggoner, Mikhail Spivakov

**Affiliations:** 1 MRC Laboratory of Medical Sciences, London, UK; 2 Institute of Clinical Sciences, Faculty of Medicine, Imperial College London, London, UK; 3 VIB, Center for Molecular Neurology, Antwerp, Belgium; 4 Faculty of Pharmaceutical, Biomedical and Veterinary Sciences, University of Antwerp, Antwerp, Belgium; 5 VIB, Center for AI and Computational Biology, Leuven, Belgium; 6 Trinity Hall, University of Cambridge, Cambridge, UK; 7 Department of Internal Medicine, Erasmus MC University Medical Center, Rotterdam, the Netherlands; 8 Immunology Graduate Program, University of Cincinnati College of Medicine, Cincinnati, OH USA; 9 Medical Scientist Training Program, University of Cincinnati College of Medicine, Cincinnati, OH USA; 10 Division of Human Genetics and Center for Autoimmune Genomics and Etiology (CAGE), Cincinnati Children’s Hospital Medical Center, Cincinnati, OH USA; 11 Department of Microbial Infection and Immunity, The Ohio State University, Columbus, OH USA; 12 Pelotonia Institute for Immuno-Oncology, The Ohio State University, Columbus, OH USA; 13 Division of Immunobiology, Cincinnati Children’s Hospital Medical Center, Cincinnati, OH USA; 14 Division of Rheumatology, Cincinnati Children’s Hospital Medical Center, Cincinnati, OH USA; 15 Center for Genetics and Rare Diseases, Sanford Research, Sioux Falls, SD USA; 16 University of Cambridge, Cambridge, UK; 17 Department of Haematology, University of Cambridge, Cambridge, UK; 18 National Health Service (NHS) Blood and Transplant, Cambridge, UK; 19 Department of Clinical and Biomedical Sciences, Faculty of Health and Life Sciences, University of Exeter Medical School, Exeter, UK; 20 The Babraham Institute, Cambridge, UK; 21 Department of Biological Sciences, Florida State University, Tallahassee, FL USA; 22 Department of Pediatrics, University of Cincinnati College of Medicine, Cincinnati, OH USA; 23 Division of Developmental Biology, Cincinnati Children’s Hospital Medical Center, Cincinnati, OH USA; 24 Division of Biomedical Informatics, Cincinnati Children’s Hospital Medical Center, Cincinnati, OH USA; 25 Division of Allergy and Immunology, Cincinnati Children’s Hospital Medical Center, Cincinnati, OH USA; 26 Department of Otolaryngology, Head and Neck Surgery, University of Cincinnati College of Medicine, Cincinnati, OH USA; 27 Divisions of Pediatric Otolaryngology and Pulmonary & Sleep Medicine, Cincinnati Children’s Hospital Medical Center, Cincinnati, OH USA; 28 Department of Metabolism, Digestion and Reproduction, Imperial College London, London, UK; 29 MRC Biostatistics Unit, Cambridge Biomedical Campus, Cambridge Institute of Public Health, Cambridge, UK; 30 Cambridge Institute of Therapeutic Immunology & Infectious Disease (CITIID), Jeffrey Cheah Biomedical Centre, Cambridge Biomedical Campus, University of Cambridge, Cambridge, UK; 31 Present Address: Eli Lilly and Company, Indianapolis, IN USA; 32 Present Address: Section of Pediatric Rheumatology, Department of Pediatrics, Wake Forest School of Medicine, Winston-Salem, NC USA; 33 Present Address: Centre for Haemato-Oncology, Barts Cancer Institute, Queen Mary University of London, London, UK; 34 Present Address: NeoGenomics, Cambridge, UK; 35 Present Address: British Heart Foundation Cardiovascular Epidemiology Unit, Department of Public Health and Primary Care, University of Cambridge, Cambridge, UK; 36 Present Address: Heart and Lung Research Institute, University of Cambridge, Cambridge, UK; 37 Present Address: European Bioinformatics Institute, Hinxton, UK; 38 Present Address: Cancer Research UK Cambridge Research Institute, Cambridge, UK; 39 Present Address: University of Kent, Canterbury, UK; 40 Present Address: Laboratory for Nuclear Dynamics, Institute for Protein Research, Osaka University, Osaka, Japan; 41 Present Address: Institute of Medical Science, University of Tokyo, Tokyo, Japan

**Keywords:** Gene regulation, Computational biology and bioinformatics, Functional genomics, Immunogenetics

## Abstract

Innate lymphoid cells (ILCs) are rare tissue-resident lymphocytes that functionally mirror cells of CD4^+^ T helper lineage but lack antigen receptors. Type 3 ILCs (ILC3s) are enriched at barrier sites, regulating inflammation and promoting tissue integrity. Here we profile the promoter-anchored chromosomal contacts of primary human ILC3s using low-input, high-resolution targeted chromosome conformation capture and compare them with those in CD4^+^ T cells. We use these data to link Crohn’s disease genome-wide association study variants with target genes, implicating both known and unanticipated candidates, including *CLN3*, a causal gene for Batten disease. We show that *Cln3* overexpression in a mouse ILC3-like cell line alters stimulation-induced transcriptional programs and cytokine secretion. Extending our approach to five additional immune genome-wide association study traits reveals enrichment for regulators of ILC3 activation. Our work develops methods, maps long-range gene regulation in ILC3s, and prioritizes immune disease risk genes with roles in this clinically relevant immune cell type.

## Abstract (web summary)

High-resolution mapping of promoter-anchored chromosomal contacts in type 3 innate lymphoid cells (ILC3s), coupled with GWAS effector gene prioritization and functional studies, identifies ILC3-linked genes as potential mediators of risk for several immune diseases.

## Main

Innate lymphoid cells (ILCs) are tissue-resident lymphocytes that mirror cells of CD4^+^ T helper lineage but lack antigen receptors, responding instead to environmental cues through cytokine production and immunomodulatory activity^1,2^. Three major ILC groups have been defined based on cytokine profiles and developmental regulators^2,3^. Among these, type 3 ILCs (ILC3s) are enriched at barrier sites, including the gut, where they regulate inflammation and epithelial integrity^2–4^. ILC3-derived cytokines such as IL-17 and IL-22 promote mucosal homeostasis, and their overexpression has been linked to the development or exacerbation of Crohn’s disease^5–8^.

Immune disorders, including Crohn’s disease, are known to have a genetic component, with genome-wide association studies (GWAS) identifying hundreds of disease susceptibility variants associated with these conditions^9^. Given the importance of ILC3s in immune control, it is likely that some of these variants affect ILC3 function. However, as most GWAS variants are noncoding and these studies are, by design, cell-type agnostic, identification of causal genes and cell types implicated by GWAS variants is challenging. High-resolution chromosome conformation capture (Hi-C) techniques such as promoter capture Hi-C (PCHi-C) enable sensitive detection of promoter-anchored interactions and prioritization of target genes of noncoding GWAS variants that reside in distal regulatory regions such as enhancers^10,11^. However, it has been difficult to apply these techniques to rare cell types such as ILC3s.

Here we use a low-input PCHi-C protocol to map the three-dimensional regulatory architecture of primary human ILC3s, alongside CD4^+^ T helper cells as an abundant adaptive immune population. We link promoters to distal regulatory elements based on these data with a combination of statistical interaction detection and a PCHi-C-adapted activity-by-contact (ABC) model. To integrate the enhancer–promoter contact maps with GWAS data, we develop multiCOGS, a Bayesian gene prioritization framework incorporating summary statistics imputation and multivariate fine-mapping. This strategy identifies both known and previously unrecognised candidate genes including *CLN3*, a Batten disease-causing gene, which we validate functionally in a mouse ILC3-like cell line. We extend ILC3 PCHi-C-based multiCOGS analysis to five additional immune diseases and show that the prioritized genes are enriched for regulators of ILC3 activation. Together, our results reveal *cis*-regulatory circuitries in ILC3s and prioritize GWAS effector genes implicating this rare, clinically relevant cell type in Crohn’s disease and other immune disorders.

## Results

### A compendium of promoter-anchored chromosomal contacts in human ILC3s

**Fig. 1:**
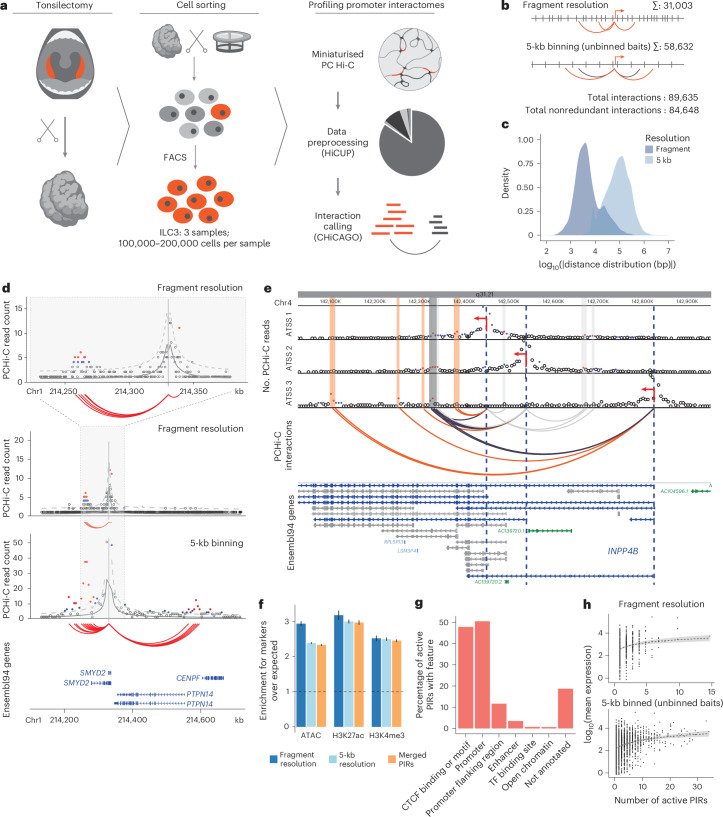
Compendium of promoter interactions in ILC3s. **a**, Outline of the study. **b**, Chromosomal interaction binning strategy. The analysis is done in two modes: fragment resolution (no binning) and 5-kb binning. In the 5-kb binning mode, the baited (captured) fragment containing a gene promoter is left unbinned to enable high-resolution linkage between the promoter and distal enhancers. Interactions uniquely detected in one mode only are shown as red arcs, and those detected in both modes are shown as gray arcs. The number of significant interactions is given for each mode individually and merged across both modes (Methods). **c**, Comparison of promoter–PIR distance distributions for PIRs detected at fragment and 5-kb resolution. **d**, Example of chromosomal interactions for the *SMYD2* gene at fragment and 5-kb resolution. The inset shows a zoomed-in view of the promoter interactions detected at fragment resolution. **e**, Example of multiple degrees of contact sharing between alternative promoters for the *INPP4B* gene. Captured alternative promoters are indicated by red arrows and blue dashed lines. The transcripts driven by these promoters (based on Ensembl 94) are shown in blue, and other *INPP4B* transcripts are shown in gray. Transcripts for processed pseudogenes are shown in light blue and long intergenic noncoding RNAs in green. PIRs are categorized as fully shared between alternative promoters (dark gray arcs), partially shared (light gray arcs) or distinct (red arcs). **f**, Enrichment of PIRs for markers of active enhancers and promoters (H3K27ac and H3K4me3) and accessible chromatin (ATAC) in ILC3s. Bars represent fold enrichment versus distance-matched background, with error bars denoting 95% confidence intervals from CHiCAGO permutation tests. **g**, Annotation of PIRs bearing active histone marks and/or ATAC-seq peaks in ILC3s (‘active PIRs’) according to the ERB. **h**, Associations between gene expression and numbers of regulatory elements identified in CHiCAGO PIRs at fragment and 5-kb resolution (leaving the baited fragments unbinned). The dashed line reflects a generalized additive model fit (smooth term estimate e.d.f. = 1 and 1.96, respectively; *P* < 2 × 10^−16^) and the gray ribbon its confidence interval. FACS, fluorescence-activated cell sorting.

To profile promoter-anchored chromosomal contacts in ILC3s, we used our low-input *DpnII*-based PCHi-C protocol^12,13^ in ILC3s isolated from human tonsils (Fig. 1a). Significant promoter contacts were detected with CHiCAGO^14^ at single-fragment resolution, as well as after pooling ‘other end’ fragments into ~5-kb bins, while leaving the baited promoter-containing *DpnII* fragment unbinned. Using this approach, we detected 31,003 contacts between promoters and promoter-interacting regions (PIRs) at single-fragment resolution and 58,632 contacts in 5-kb bins (Fig. 1b and Supplementary Table 1; Data S1 and S2 at OSF^15^). Binning resulted in detection of longer-range contacts, as we reported previously in other cell types^16^ (Fig. 1c,d). For further analyses, we merged PIR sets detected at both resolutions (Methods).

**Extended Data Fig. 1:**
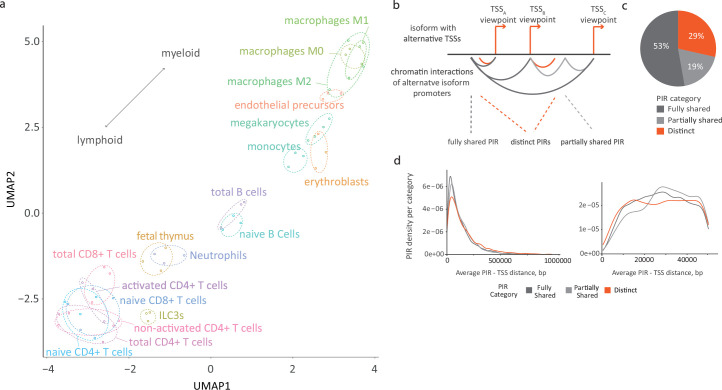
Compendium of promoter-enhancer interactions in ILC3s. **a**, UMAP of CHiCAGO scores detected for PCHi-C in ILC3s versus public data in 17 primary human blood cell types^17^. **b**, Scheme representing the classification of PIRs detected at alternative transcription start sites (ATSSs) of the same gene: ‘fully shared’ (shared across all captured ATSSs), partially shared and distinct (unique to a single ATSS). **c**, Pie chart showing the degree of enhancer sharing across alternative transcription start sites (ATSSs) for short-range contacts. **d**, Distance distribution of ATSS-specific and shared PIRs at 5-kb binned (baits unbinned) resolution. Left plot shows interactions up to 1 Mb (Kruskal-Wallis test *P* < 2.22 × 10^−16^; pairwise Wilcoxon test *P* = 8.68 × 10^−6^ (partially shared vs. fully shared), *P* = 4.46 × 10^−8^ (partially shared vs. distinct) and *P* < 2.22 × 10^−16^ (fully shared vs. distinct)). Right plot shows interactions up to 50 kb (Kruskal-Wallis test *P* = 7.65 × 10^−5^; pairwise Wilcoxon test *P* = 9.8 × 10^−5^ (partially shared vs. fully shared), *P* = 6 × 10^−4^ (partially shared vs. distinct) and *P* = 1 (fully shared vs. distinct)).

A joint UMAP embedding of ILC3 promoter interaction profiles with those detected in 17 abundant blood cell types using *HindIII-*based PCHi-C^17^ segregated ILC3s with other lymphoid cell types, consistent with patterns of promoter interactions reflecting lineage history (Extended Data Fig. 1a; Data S3 at OSF^15^; Methods). In total, more than half of PIRs (52.2%, 16,087 of 30,830) contacted at least one promoter in both ILC3s and another blood cell type (Data S3 at OSF^15^). Notably, the increased resolution afforded by using four-base cutter *DpnII* rather than six-base cutter *HindIII* enabled capture of alternative transcription start sites for 6,789 genes on separate baited fragments. Many of these genes displayed distinct interaction landscapes across isoforms (Extended Data Fig. 1b–d), as exemplified by the interaction patterns of three *INPP4B* alternative transcription start sites (Fig. 1e).

We next used public epigenetic data in human tonsil-derived ILC3s^18^ to annotate PIR chromatin states. As expected, ILC3 PIRs were enriched for markers of accessible and/or active enhancers (assay for transposase-accessible chromatin (ATAC) peaks and histone H3 lysine 27 acetylation (H3K27ac)) and active transcription (histone H3 lysine 4 trimethylation (H3K4me3)), with 18,231 PIRs bearing combinations of these marks (‘active PIRs’; Fig. 1f). Nearly two-thirds of active PIRs (65.9%, 12,021 of 18,231) overlapped with active regulatory regions annotated in the Ensembl Regulatory Build (ERB) (‘promoter’, ‘promoter flanking regions’, ‘open chromatin’; Methods)^19^, and 47.5% (8,658 of 18,231) overlapped with ERB-annotated CTCF binding sites (Fig. 1g). However, only 3% (636 of 18,231) of these PIRs overlapped with ERB-annotated enhancers^20^, and nearly 20% (3,425 of 18,231) did not overlap with any ERB annotations (Fig. 1g).

Finally, to probe the relationship between enhancer–promoter connectivity and gene expression, we integrated these data with publicly available single-cell RNA sequencing (RNA-seq) profiles of human mucosal tissue ILC3s^21^. In agreement with studies in other cell types^17^, we observed significant positive associations between numbers of active PIRs and gene expression, at both fragment and 5-kb resolution (Fig. 1h).

### Enhancer–promoter contacts identified by ABCC complement significant promoter interactions

To further increase the sensitivity of detection of functional enhancer–promoter chromosomal interactions, we adapted the ABC approach^22^ to PCHi-C data. In contrast to CHiCAGO, which detects statistically significant interactions relative to a distance-dependent background, ABC, which was originally developed for Hi-C, considers any observed contact frequency between a chromatin region and a promoter to be functionally meaningful. In addition, whereas CHiCAGO scores are independent of enhancer activity levels at PIRs, ABC scores incorporate both contact frequency and enhancer activity^22^.

**Fig. 2:**
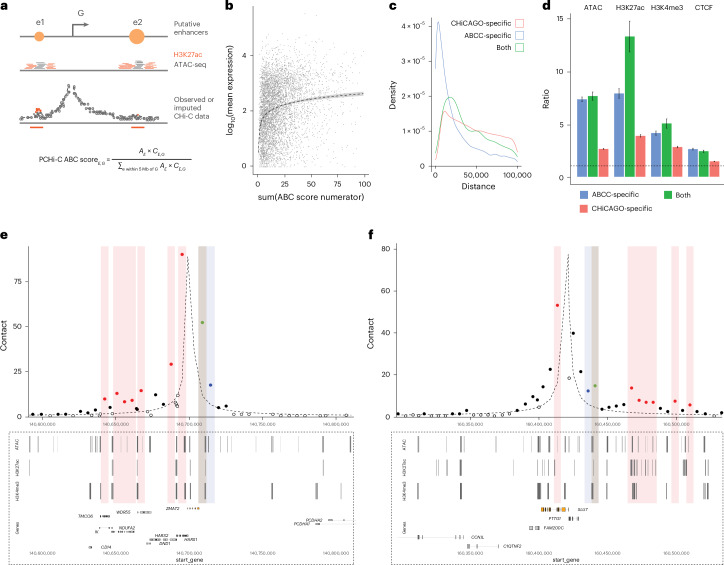
Combining ABCC and CHiCAGO to link distal elements with target genes. **a**, Schematic depicting adaptation of the ABC model for use with PCHi-C data, termed ABCC. **b**, Associations between gene expression and ABC numerator score summed across all predicted enhancers per gene. The dashed line shows a generalized additive model fit (smooth term estimate e.d.f. = 2.485, *P* < 2 × 10^−16^) and the gray ribbon its confidence interval. **c**, Interaction distance comparison across CHiCAGO-specific, ABCC-specific and shared interactions. **d**, Enrichment for markers of active and accessible chromatin within CHiCAGO-specific, ABCC-specific and shared PIRs. Bars represent fold enrichment versus distance-matched background, with error bars denoting 95% confidence intervals from CHiCAGO permutation tests. **e**,**f**, Representative examples of CHiCAGO- and ABCC-detected contacts for *ZMAT2* (**e**) and *SLU7* (**f**) promoters. The dashed line shows expected counts estimated using the CHiCAGO distance function. PIRs detected with CHiCAGO at 5-kb resolution are shown as red dots and shading, those detected with ABCC as blue dots and shading, and those detected by both approaches as green points and shading. Black-filled dots represent imputed counts considered by ABCC, corresponding to the maximum value between observed and expected counts. Unfilled dots represent observed counts falling below expected values.

**Extended Data Fig. 2:**
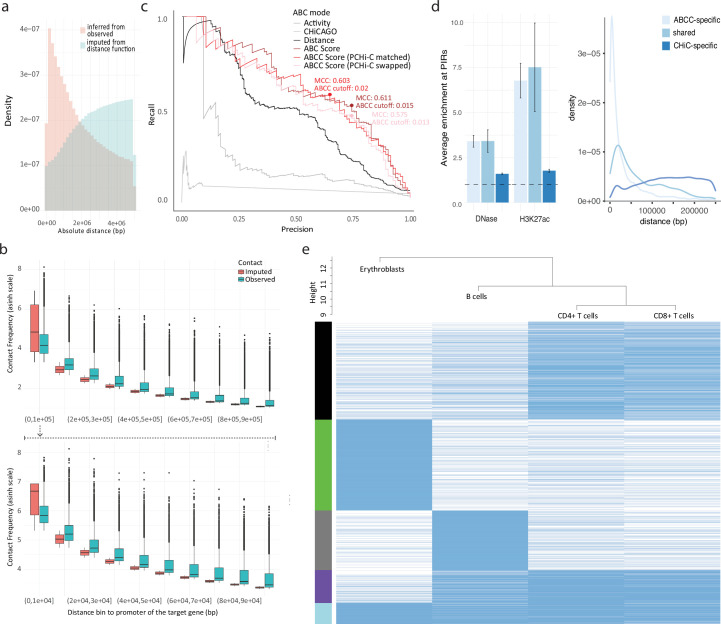
Benchmarking of the ABCC approach with public data. **a**, Density distribution of promoter interactions inferred from observed PCHi-C contact frequencies (pink) and those imputed using the CHiCAGO distance function (cyan) across genomic interaction distances. **b**, Contact frequency distributions stratified by distance. Observed PCHi-C contacts are shown in green; imputed contacts (using expected frequencies estimated using the CHiCAGO distance function) are shown in blue. Similarly to standard ABC, frequency capping is introduced for short-range imputed contacts (<5 kb). **c**, Precision–recall curves benchmarking the predictive performance of different scoring approaches for enhancer–promoter interactions in erythroblasts. Curves compare the scoring across: CHiCAGO-detected contacts, Activity alone, Distance alone, the conventional ABC score, and PCHi-C-based ABCC score in two modes: ‘matched’ (using PCHi-C cell-type specific profile for erythroid cells) and ‘swapped’ (in which a PCHi-C dataset with a similar read coverage from a different cell type, CD4 + T cells, is used instead). MCC, Matthews correlation coefficient, an alternative to the AUC metric that is more informative under class imbalance and more sensitive to performance at a fixed decision threshold^113^. **d**, Enrichment of epigenetic marks at PIRs: DNase marking chromatin accessibility and H3K27ac marking active enhancers (left plot) and distance distribution of ABCC-specific, PCHiC-specific and shared enhancer-promoter links (right plot) in K562 cells for 0.023 ABCC threshold. **e**, Hierarchical clustering heatmap of enhancer–promoter interactions predicted with ABCC across cell types (erythroblasts, B cells, CD4 + T helper cells, CD8 + T cells).

In our ABC adaptation, termed activity-by-captured-contact (ABCC), the contact frequencies are estimated from PCHi-C data that are imputed based on CHiCAGO-generated statistical models (Fig. 2a, Extended Data Fig. 2a,b and Methods). We validated ABCC using public enhancer perturbation data^22^ and confirmed that this approach could accurately identify functional enhancer–promoter interactions in a lineage-specific manner and provide regulatory information complementary to CHiCAGO-detected contacts (Extended Data Fig. 2 and Supplementary Note).

We applied ABCC to ILC3 PCHi-C data, producing 18,877 putative enhancer–promoter pairs across 17,690 genes (Data S4 at OSF^15^). Similar to the findings for CHiCAGO-defined PIRs, we observed positive associations between numbers of ABCC-linked enhancers per gene and gene expression (Fig. 2b). However, ABCC interactions generally spanned shorter distances than CHiCAGO-detected pairs (median distance ~69 kb versus ~108 kb, respectively; Wilcoxon rank-sum test *P* < 2.2 × 10^−16^; Fig. 2c), and the two sets of contacts showed limited overlap (8.4%; Data S5 at OSF^15^). As expected from their definition, ABCC-linked enhancers were strongly enriched for features of active or accessible chromatin (Fig. 2d). They also showed enrichment for CTCF binding sites comparable to that of CHiCAGO-defined PIRs (Fig. 2d).

For downstream analyses, we combined ABCC- and CHiCAGO-defined promoter contacts (referred to collectively as PIRs hereafter). Representative examples of the regulatory landscapes detected by both approaches are shown in Fig. 2e,f.

### Promoter interactomes in ILC3s and CD4^+^ T cells reveal shared and differential regulatory circuits

ILC3s have developmental similarities^23,24^ and share core ‘immune modules’ with CD4^+^ T helper cells^24–26^; this prompted us to use this abundant cell type for comparative analysis and identification of ILC3-specific regulatory circuits. To this end, we generated and processed high-resolution PCHi-C data for CD4^+^ T cells using the same protocol, identifying 31,252 and 87,348 interactions at single-fragment and 5-kb resolution, respectively (Data S6 and S7 at OSF^15^). In addition, we detected 30,258 enhancer–gene pairs in CD4^+^ T cells with ABCC across 16,956 genes (Data S8 and S9 at OSF^15^), 30% of which were shared with ABCC pairs identified in ILC3s.

**Fig. 3:**
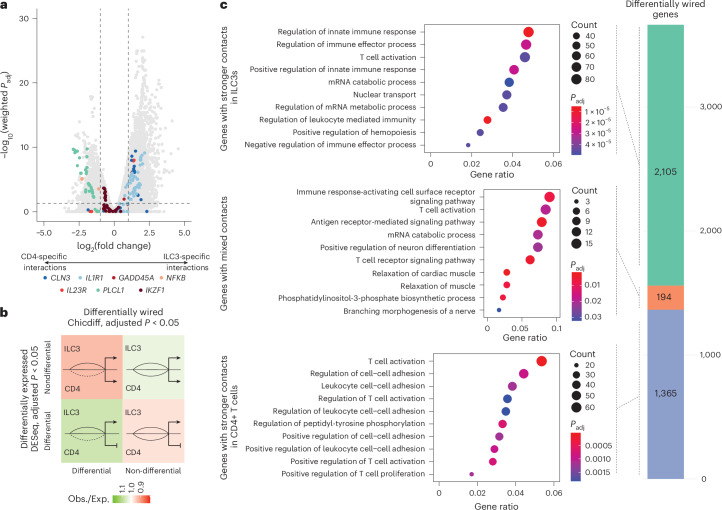
Differential enhancer–promoter interactions between ILC3s and CD4^+^ T cells. **a**, Volcano plot of differential interactions between ILC3s and CD4^+^ T cells detected by Chicdiff^27^, highlighting those of selected immune-related genes (*CLN3*, *IL1R1*, *GADD45A*, *NFKB*, *IL23R*, *PLCL1*, *IKZF1*). Statistical significance was evaluated using two-sided Wald test within a negative binomial generalized linear model framework, with *P* values adjusted for multiple testing across all interactions using independent hypothesis weighting^84^. **b**, Relationship between differential expression (DESeq2, adjusted *P* < 0.05) and differential wiring of promoter contacts (Chicdiff, adjusted *P* < 0.05). Statistical significance was determined using Pearson’s chi-squared test with Yates’ continuity correction (*χ*^2^ = 23.938, d.f. = 1, exact *P* = 9.95 × 10^−7^). **c**, Gene ontology (GO) enrichment analysis of genes with stronger contacts in ILC3s (top), CD4^+^ T cells (bottom) or a mixture of contacts that were stronger in either cell type (middle), showing biological processes related to immune cell activation, adhesion and differentiation. Pathway enrichment was assessed using one-sided hypergeometric test, with *P* values adjusted globally for multiple comparisons using the Benjamini–Hochberg false discovery rate procedure (adjusted *P* < 0.05 was considered to indicate significance; full results are listed in Supplementary Tables 2–5). Bubble size reflects the number of genes; color indicates adjusted *P* values. The bar plot shows the overlap between differentially wired genes (as evaluated by Chicdiff) in ILC3s and CD4^+^ T cells. *P*_adj_, adjusted *P* value.

**Extended Data Fig. 3:**
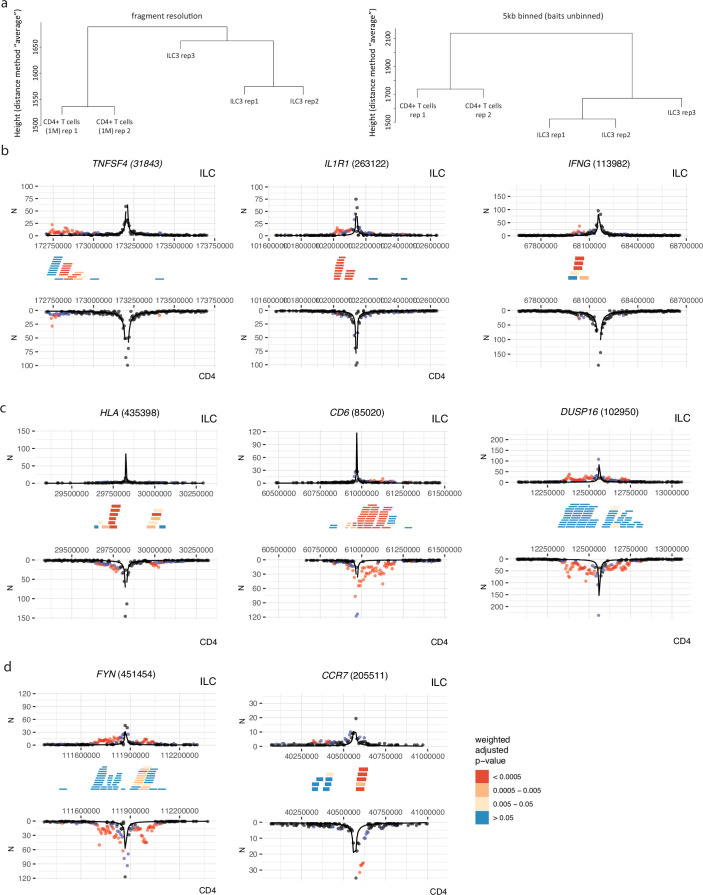
Genes with differential contacts in ILC3s and CD4 + T cells. **a**, Hierarchical clustering of CHiCAGO signals based on data in ILC3s and CD4 + T cells. **b**-**d**, Examples of captured promoters with differential wiring between ILC3s and CD4 + T cells: promoters with stronger (**b**) and weaker (**c**) contacts in ILC3s compared with CD4 + T cells, as well as with contacts in both cell types (**d**).

Differential analysis of promoter interactions between ILC3s and CD4^+^ T cells with Chicdiff^27^ revealed a total of 19,038 cell-type-specific contacts across 3,664 genes (1,818 at fragment resolution and 17,220 at 5-kb resolution; weighted adjusted *P* < 0.05; Fig. 3a and Extended Data Fig. 3). As expected, we also detected a significant association between differential interactions and differential expression (chi-squared test *P* = 9.95 × 10^−7^) (Fig. 3b). Pathway enrichment analysis revealed immune regulatory programs associated with ILC3-specific and CD4^+^ T cell-specific contacts (Fig. 3c, Supplementary Tables 2–5 and Supplementary Note). Overall, this comparative analysis highlighted both shared and distinct *cis*-regulatory wiring of ILC3s and CD4^+^ T cells, reflecting their specialized roles in innate versus adaptive immune responses and coordinated regulation of immune activation pathways.

### PIRs in ILC3s and CD4^+^ T cells are enriched for immune disease-associated variants

**Fig. 4:**
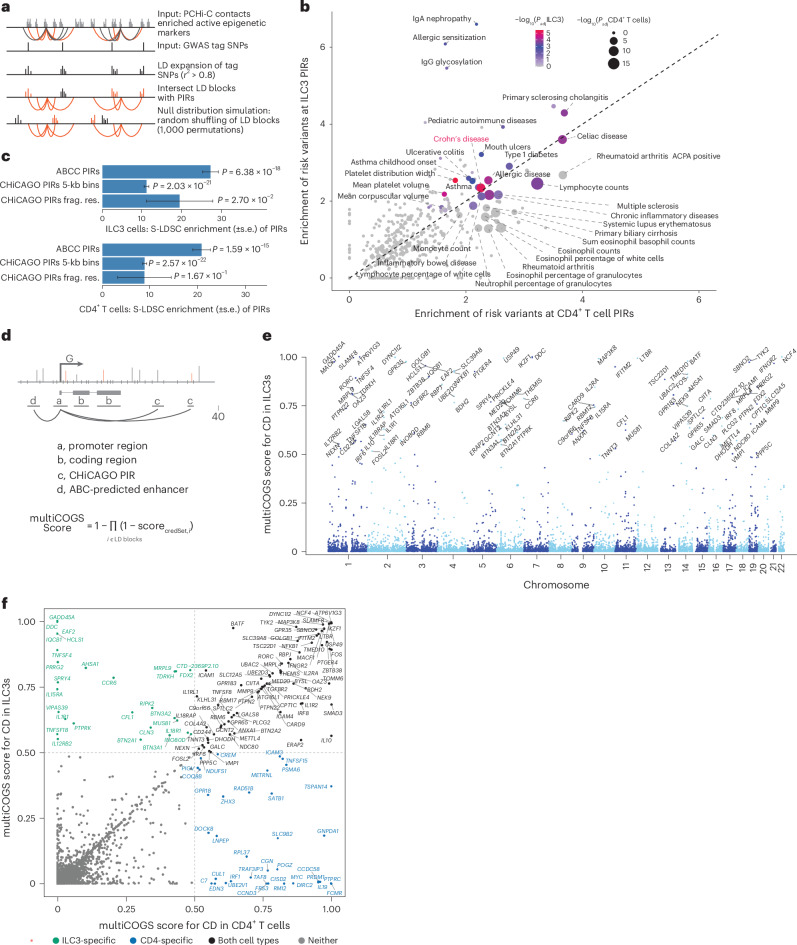
Statistical integration of PCHi-C results for ILC3s and CD4^+^ T cells with GWAS enables gene prioritization for Crohn’s disease. **a**, Schematic of the RELI algorithm used to estimate enrichment of genetic risk loci within PIRs. **b**, Enrichment of risk variants in ILC3 versus CD4^+^ T cell ‘active’ PIRs across 495 diseases and traits, determined using the RELI method^28^. *P* values were adjusted for multiple testing using the Bonferroni procedure. Traits with adjusted *P* values < 0.05 in ILC3s and/or CD4^+^ T cells are labeled. **c**, Stratified LD score regression analysis (using LDSC^85^) for enrichment of Crohn’s disease risk heritability at PIRs of ILC3s and CD4^+^ T cells. Bars show LDSC-estimated enrichment coefficients for each PIR category, and error bars show the standard errors reported by the model. Statistical significance was determined using one-sided *z*-test relative to the baseline model; exact *P* values are reported next to each bar. **d**, Schematic of the multiCOGS algorithm. **e**, Manhattan plot of multiCOGS gene prioritization scores for Crohn’s disease risk based on integration of GWAS with promoter interactions in ILC3s. Genes with multiCOGS scores greater than 0.5 are labeled. **f**, Distribution of multiCOGS gene scores for Crohn’s disease computed based on PCHi-C data in ILC3s and CD4^+^ T cells. Genes are colored depending on whether they exceeded a multiCOGS score cutoff of 0.5 in ILC3s only (green), CD4^+^ T cells only (blue) or both cell types (black). The rest of the genes are shown as gray dots. Frag. res., fragment resolution.

We used the RELI algorithm^28^ to detect enrichment of active PIRs for variants associated with 495 healthy and pathological GWAS traits listed in the GWAS Catalog^29^ (Fig. 4a and Methods).

**Extended Data Fig. 4:**
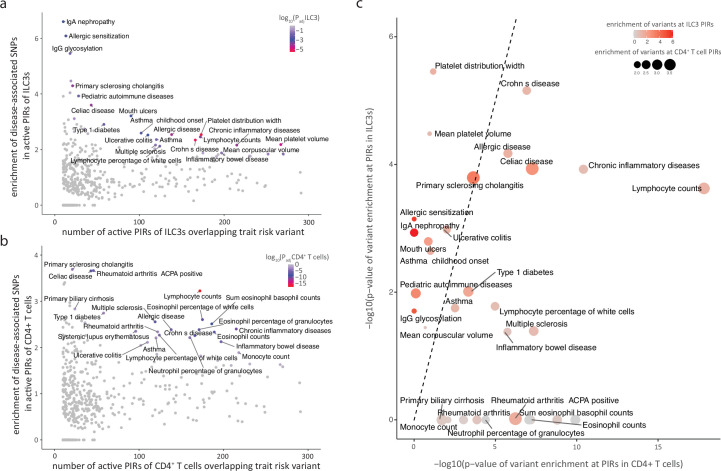
Additional information for the RELI analysis of risk loci enriched in ILC3 and CD4 + T-cell PIRs. **a**,**b**, RELI enrichment of risk variants in ILC3s (**a**) and CD4s active PIRs (**b**) across 495 diseases and traits. **c**, Adjusted *P*-value of RELI enrichment of risk variants ILC3s vs. CD4s active PIRs across 495 diseases and traits. In all panels, traits with Bonferroni-adjusted *P*-value  < 0.05 in the respective cell type(s) are labelled.

In ILC3s, RELI revealed significant enrichment for variants associated with 21 of 495 traits (Bonferroni-adjusted *P* < 0.05; Fig. 4b, Extended Data Fig. 4a and Supplementary Table 6). Autoimmune diseases (EFO:0005140) affecting a broad array of tissues and organs where ILC3s are known to reside were overrepresented among these traits (one-tailed hypergeometric test, *P =* 1.08 × 10^−^^5^). These tissues and organs included the gut (Crohn’s disease, celiac disease, ulcerative colitis, primary sclerosing cholangitis), airways (asthma, hay fever) and central nervous system (multiple sclerosis). We also noted enrichment for several peripheral blood cell traits, including platelet width, lymphocyte count and corpuscular volume (Supplementary Table 6).

In CD4^+^ T helper cells, RELI detected significant enrichment for variants associated with 22 of 495 traits (Bonferroni-adjusted *P* < 0.05) (Extended Data Fig. 4b and Supplementary Table 7). There was a strong correlation between the traits enriched in the two cell types (*R*^2^ = 0.85, correlation test *P* = 5.2 × 10^−4^) (Fig. 4b and Extended Data Fig. 4c), consistent with their overlapping *cis*-regulatory circuits. However, several traits displayed cell type specificity; these included allergic sensitization, mouth ulcers and IgG glycosylation in ILC3s, and primary biliary cirrhosis, rheumatoid arthritis and systemic lupus erythematosus in CD4^+^ T cells (Fig. 4b, Extended Data Fig. 4c and Supplementary Table 7).

### multiCOGS links imputed, fine-mapped Crohn’s disease GWAS signals to candidate effector genes through promoter contacts in ILC3s and CD4^+^ T cells

RELI analysis identified significant enrichment of Crohn’s disease risk variants within active PIRs in both ILC3s and CD4^+^ T helper cells (~2.3-fold; Bonferroni-adjusted *P* = 6.96 × 10^−6^ and *P* = 1.19 × 10^−7^, respectively), which we confirmed using stratified linkage disequilibrium (LD) score regression (Fig. 4c). Given the importance of both cell types in the pathogenesis of Crohn’s disease^30–34^, we next sought to prioritize effector genes linked to Crohn’s disease risk variants in these cells using our PCHi-C data. To this end, we developed multiCOGS, a Bayesian prioritization algorithm that scores the association of each gene with a trait based on the location of fine-mapped GWAS signals within gene coding regions, gene promoters and PIRs (Fig. 4d and Methods). multiCOGS extends our previously published COGS^17,35^ framework by incorporating summary-statistics-based imputation^36^ and multivariate fine-mapping approaches such as SuSiE^37–39^ to increase the accuracy and coverage of gene prioritization.

**Extended Data Fig. 5:**
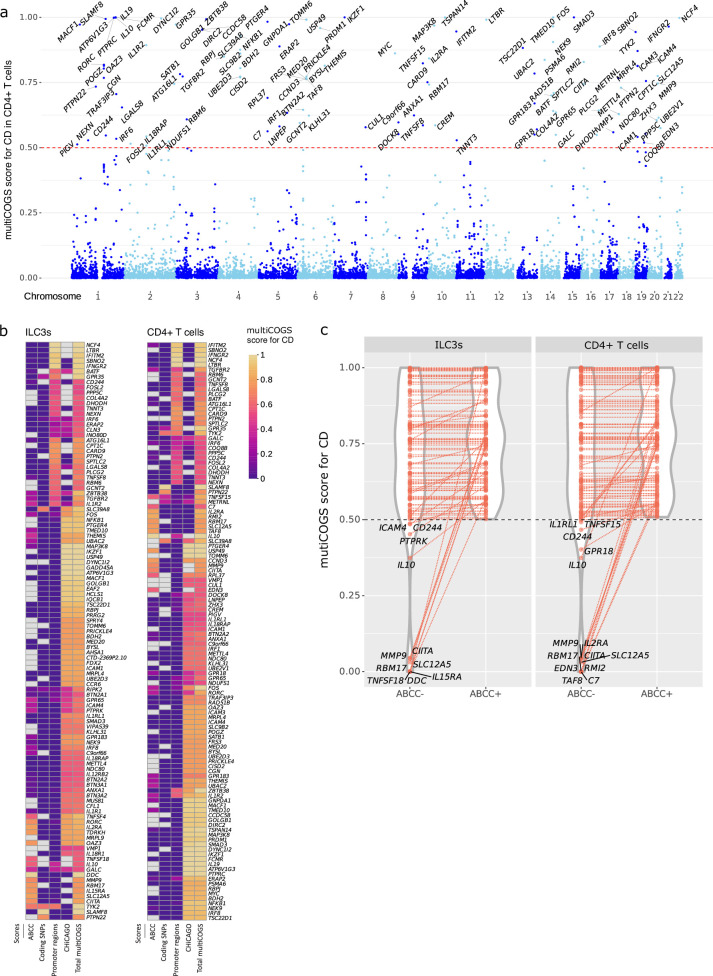
MultiCOGS prioritizes candidate gene sets for Crohn’s disease risk. **a**, Manhattan plot showing multiCOGS scores for Crohn’s disease (CD) risk based on promoter contacts in CD4 + T cells. **b**, Heatmaps of region contributions to multiCOGS scores in ILC3s and CD4 + T cells in CD. **c**, Illustration of genes that were only prioritized for CD with the addition of ABCC, in ILC3s and CD4 + T cells. In each graph, the multiCOGS score with and without ABCC is plotted for all genes that were prioritized in the full multiCOGS run (score > 0.5 with ABCC).

**Extended Data Fig. 6:**
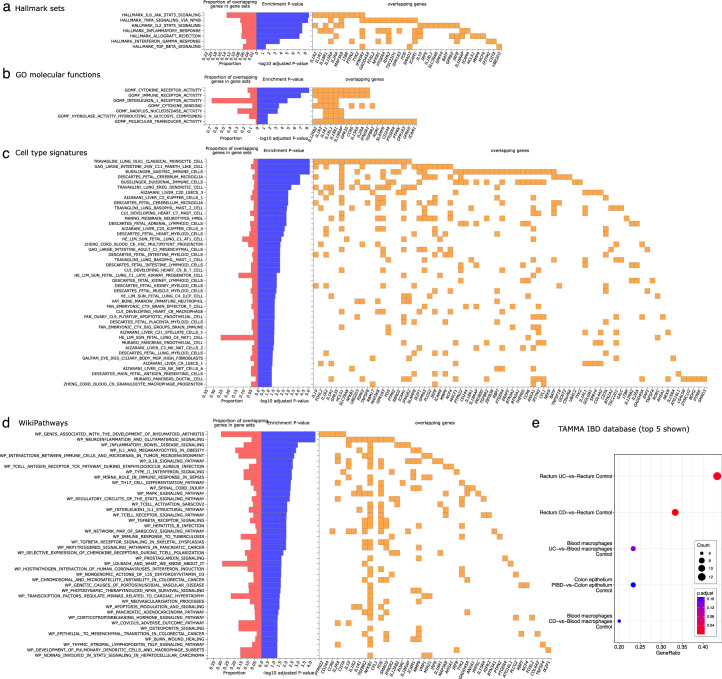
Biological annotation of multiCOGS-prioritized Crohn’s disease effector genes in ILC3s. **a-d**, Enriched gene sets among multiCOGS genes detected using the GENE2FUNC pipeline in FUMA^86^ for the following databases: MSigDB hallmark sets (**a**), GO molecular functions (**b**), MSigDB cell type signatures (**c**), MSigDB WikiPathways (**d**). **e**, Enrichment analysis for differentially expressed gene sets among multiCOGS genes in the TAMMA IBD database.

**Extended Data Fig. 7:**
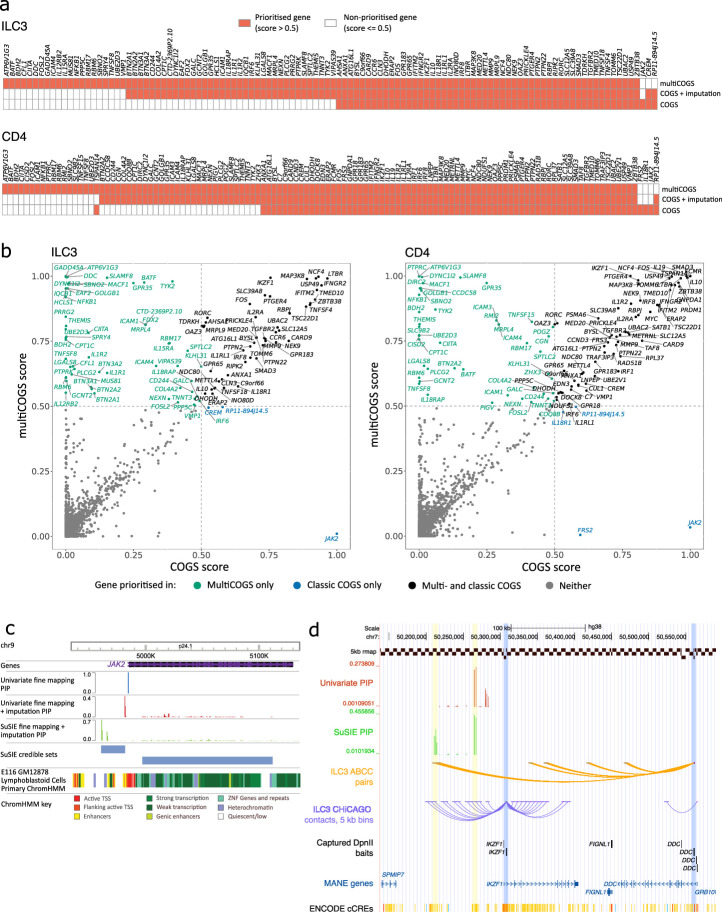
Comparison of gene prioritization for Crohn’s disease by classic COGS versus multiCOGS. **a**, Comparison of prioritized gene sets between classic COGS, classic COGS plus imputation, and multiCOGS (that is, imputation plus multivariate fine mapping, processed via the multiCOGS algorithm) for Crohn’s disease (CD). Shown for ILC3s and CD4 + T cells. **b**, Comparison of COGS scores and multiCOGS scores for genes in ILC3 cells (left) and CD4 + T cells (right) for Crohn’s disease. Green labels indicate genes prioritized in multiCOGS only, blue in classic COGS only, and black in both. **c**, Plot of the *JAK2* locus showing the shift of the most likely causal variant from the promoter of *JAK2* to a region around 20 kb upstream of the promoter upon multivariate fine mapping, leading to a lower multiCOGS vs. classic COGS score. No chromosomal interactions were observed between this region and the *JAK2* promoter in ILC3 or CD4 + T cells. **d**, Illustration of multiCOGS prioritization of *IKZF1* and *DDC* in ILC3s at the chr7p locus. At this locus, multivariate fine mapping identifies two credible sets of variants (yellow vertical bars), whereas univariate fine mapping only detects one. PCHi-C interactions connect these likely causal variants to the *IKZF1* promoter (first blue vertical bar). However, ABCC interactions also connect one of the credible sets to the DDC promoter (second blue vertical bar). Thus, multiCOGS prioritises both genes, whereas classic COGS prioritises only *IKZF1*. PIP; posterior inclusion probability.

We applied multiCOGS to a Crohn’s disease GWAS meta-analysis^40^ using CHiCAGO- and ABCC-defined PIRs in ILC3s and CD4^+^ T cells. At the established COGS score cutoff of 0.5 (ref. ^17^), multiCOGS prioritized 109 genes in ILC3s (Fig. 4e) and 118 genes in CD4^+^ T cells (Extended Data Fig. 5a and Supplementary Tables 8 and 9), mostly through linking of noncoding, likely causal variants to gene promoters via CHiCAGO-defined contacts (Extended Data Fig. 5b). Notably, ABCC contributed to only ~11% of prioritized genes in both cell types (Extended Data Fig. 5c). The prioritized candidates had known functions in cytokine signaling (*IL10*, *IL1RL1*, *IL2RA*, *IFNGR2*), autophagy (*ATG16L1*, *GPR65*), antimicrobial responses (*PTPN2*, *IRF8*)^41,42^ and transcriptional regulation (*FOS*, *TSC22D1*, *RBPJ*) and were enriched in immune pathways that have been implicated in inflammatory bowel disease (IBD)^43–45^, including the IL6–JAK–STAT3, TNF–NF-κB and IL-23/T helper 17 cell pathways (Extended Data Fig. 6, Supplementary Table 10 and Supplementary Note). Comparison with standard COGS showed that multiCOGS substantially expanded and refined the prioritized genes (Extended Data Fig. 7, Supplementary Table 11 and Supplementary Note). Notably, at several loci, multiCOGS prioritized multiple candidates through distinct credible sets. For example, at chr7p, both COGS and multiCOGS prioritized *IKZF1* (encoding the Ikaros transcription factor (TF)^46^) based on CHiCAGO-defined contacts. However, multiCOGS also prioritized DOPA decarboxylase gene *DDC*, which encodes a potential regulator of immune cell infiltration^47^, based on a separate credible set overlapping ABCC-linked enhancers (Extended Data Fig. 7d).

We searched for prior evidence of association of multiCOGS-prioritized genes with Crohn’s disease (or, more broadly, IBD) by querying them in OpenTargets, curated gene-to-disease databases and functional studies^48–52^. According to these sources, more than half of the genes prioritized in ILC3s (61 of 109) and CD4^+^ T cells (67 of 118) had not previously been implicated in IBD (Supplementary Table 11). In particular, 20 of the 29 genes selectively prioritized in ILC3s and not CD4^+^ T cells (Fig. 4f) had not previously been linked to IBD in the studied datasets. This set included genes such as *GADD45A* and *PTPRK* with known roles in immunity and T cell development^53,54^ and some with unexpected functions, including *DDC* as mentioned above, as well as *CLN3*, which encodes a lysosomal protein whose mutations cause the neurodegenerative Batten disease^55^.

**Fig. 5:**
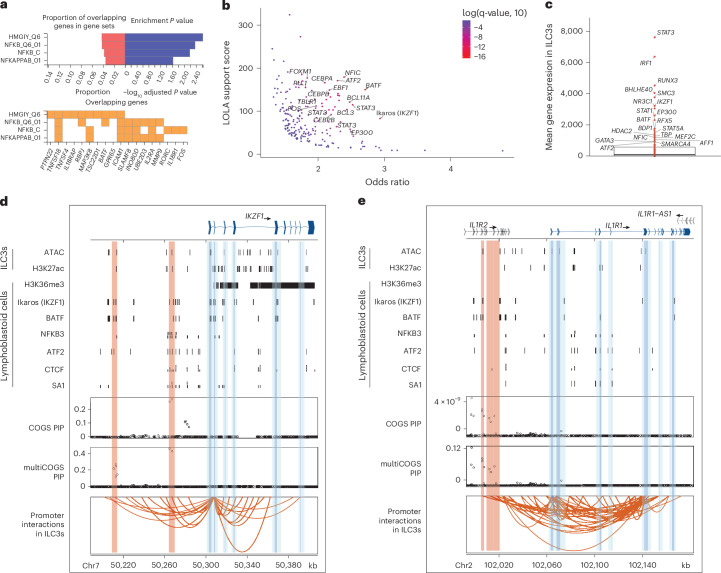
Characterization of genes associated with Crohn’s disease risk prioritized by multiCOGS in ILC3s and their putative TF regulators. **a**, Significant sets of multiCOGS-prioritized genes predicted to bind specific TFs in their promoter regions, according to the MSigDB TF targets database, detected using the GENE2FUNC pipeline in FUMA (hypergeometric overrepresentation test)^86^. TF sets are labeled (rows), with the proportions of all multiCOGS genes per set and associated *P* values (adjusted for multiple testing via the Benjamini–Hochberg method) shown in the top panel and gene names in the bottom panel. **b**, Enrichment analysis for TF binding sites at active PIRs for genes prioritized by multiCOGS versus active PIRs of all genes submitted to multiCOGS analysis. **c**, Expression of TFs enriched at the PIRs of prioritized genes. Outliers were removed for clarity. **d**,**e**, Examples of genes prioritized by multiCOGS for Crohn’s disease (*IKZF1* (**d**) and *IL1R1* (**e**)), showing patterns of TF binding in lymphoblastoid cell lines and posterior inclusion probability (PIP) profiles generated by the Wakefield fine-mapping procedure^87^ assuming a single causal variant per LD block used in classic COGS (‘COGS PIP’) and the SuSiE^38^ multivariate fine-mapping algorithm used in multiCOGS (‘multiCOGS PIP’). Vertical dark blue and light blue bands, respectively, highlight annotated gene promoters and promoter-proximal regions (±5 restriction fragments) considered in (multi)COGS analysis in addition to PIRs. Vertical red bands highlight PIRs harboring Crohn’s disease risk-associated SNPs with high PIPs. Orange arcs correspond to significant interactions (CHiCAGO score > 5) at 5-kb resolution for *IKZF1* and *IL1R1*, respectively.

Finally, we predicted potential transcriptional regulators of the prioritized loci in ILC3s using two complementary approaches. Gene-centric analysis identified HMGA1 and NF-κB as candidate regulators of genes prioritized in Crohn’s disease (Fig. 5a and Supplementary Table 12), whereas region-centric analysis identified enrichment of the predicted binding sites for 97 TFs within their PIRs (Fig. 5b and Supplementary Table 12). These TFs included established regulators of inflammatory response, such as Ikaros (encoded by *IKZF1*)^46^, BATF^56^ and NFKB3 (also known as RELA)^57–59^, all of which are highly expressed in ILC3s (Fig. 5c). For example, the promoter of *IKZF1* contacted two active PIRs harboring fine-mapped Crohn’s disease variants in ILC3s, with predicted occupancy of Ikaros itself, BATF, NFKB3, ATF2, CTCF and SA1 inferred from lymphoblastoid cell line data (Fig. 5d). Similarly, the promoter of *IL1R1*, which encodes an ILC3-activating receptor^60^, contacted active PIRs harboring Crohn’s disease-associated variants and showing evidence of Ikaros, BATF and CTCF binding based on lymphoblastoid cell line data (Fig. 5e).

Overall, multiCOGS prioritization implicated inflammatory signaling genes and their upstream TF regulators as candidate mediators of Crohn’s disease susceptibility in ILC3s.

### CLN3 contributes to ILC3 inflammatory capacity

**Fig. 6:**
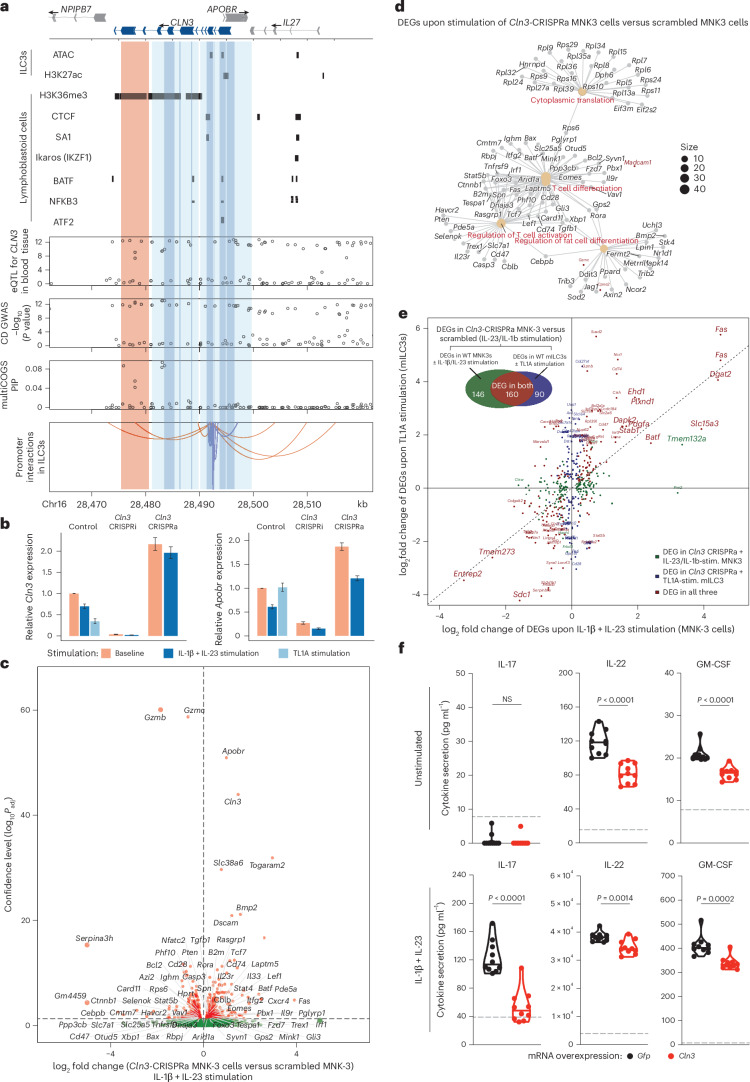
Evidence of the role of CLN3 in ILC3 inflammatory function. **a**, Interaction profile of the human *CLN3* promoter alongside the tracks of TF binding, blood expression quantitative trait loci^65^, Crohn’s disease GWAS^40^ −log_10_ *P* values and SuSiE PIP values. Dark blue and light blue bands, respectively, highlight locations of annotated *CLN3* promoters and promoter-proximal regions (±5 restriction fragments) considered by multiCOGS in addition to PIRs. Red band highlights the ILC3-specific PIR containing Crohn’s disease-associated SNPs with high PIPs. Orange and purple arcs, respectively, depict significant interactions (CHiCAGO score > 5) in ILC3s at 5-kb and single-fragment resolution. Multiple vertical lines per TF reflect separate datasets. **b**, Up- and downregulation of *Cln3* and *Apobr* upon TL1A stimulation in mouse primary ILC3s (RNA-seq data from ref. ^64^) and upon IL-23/IL-1β stimulation in *Cln3*-targeted CRISPRi and CRISPRa MNK-3 cells (RNA-seq data from this study). Bars and error bars, respectively, indicate fold-change estimates and standard errors derived from DESeq2 (ref. ^88^) negative binomial regression models based on *n* = 3 replicates per condition. **c**, Differential expression of genes in IL-23/IL-1β-stimulated *Cln3*-CRISPRa MNK-3 cells relative to scrambled gRNA controls. Red dots show differentially expressed genes (stimulated *Cln3-*CRISPRa differentially expressed genes, DESeq2 adjusted *P* < 0.05), with other genes shown as green dots. **d**, Network-style representation of GO term enrichment analysis of stimulated *Cln3-*CRISPRa differentially expressed genes. **e**, Changes in expression of stimulated *Cln3-*CRISPRa differentially expressed genes (dots) upon either IL-23/IL-1β stimulation of unperturbed MNK-3 cells or TL1A stimulation of primary mouse ILC3s (data from ref. ^64^). Genes with log_2_-fold-changes greater than 1.5 in either condition are labeled in small letters and those with such changes in both conditions in large letters. **f**, Evidence that *Cln3* overexpression decreases inflammatory cytokine secretion. MNK-3 cells were electroporated with *GFP* mRNA (black) or *Cln3–Myc* mRNA (red) and then cultured either unstimulated (top row) or stimulated with IL-1β and IL-23 (bottom row) for 24 h. Cytokine concentrations (IL-17, IL-22, GM-CSF) in culture supernatants were quantified by enzyme-linked immunosorbent assay. Each point represents an individual biological replicate (*n* = 10 per condition). Data shown are from one representative experiment of three independent experiments performed. The dotted line indicates the lower limit of quantification for each assay. Statistical significance was assessed using unpaired two-sided Welch’s *t*-test. DEGs, differentially expressed genes; NS, not significant; WT, wild type.

We next focused on *CLN3*, a Batten disease gene, which was selectively prioritized for Crohn’s disease risk in ILC3s but not CD4^+^ T cells and not previously linked to any immune-mediated diseases according to our database analysis. Examination of the SuSiE fine-mapped Crohn’s disease GWAS locus underlying the prioritization of *CLN3* revealed a credible set of variants overlapping with two regions considered by multiCOGS, which were colocalized with expression quantitative trait loci for *CLN3* and nearby genes including *APOBR* (Fig. 6a and Supplementary Note).

To further investigate the role of the *CLN3* locus in ILC3s, we used mouse MNK-3 cells as a tractable model for ILC3 activation and effector function^61^. We found that *Cln3* expression was downregulated upon MNK-3 cell stimulation with IL-23 and IL-1β, cytokines that are essential for ILC3 effector function^62,63^ (Fig. 6b, left). Consistent with this observation, analysis of published RNA-seq data from primary mouse ILC3s stimulated with TL1A^64^ also showed reduced *Cln3* expression (Fig. 6b, left). Notably, adjacent gene *Apobr* was similarly downregulated under IL-23/IL-1β stimulation (Fig. 6b, right), in line with evidence of coordinated regulation of these genes in humans based on expression quantitative trait loci^65–68^. By contrast, TL1A stimulation did not affect *Apobr* expression (Fig. 6b, right).

**Extended Data Fig. 8:**
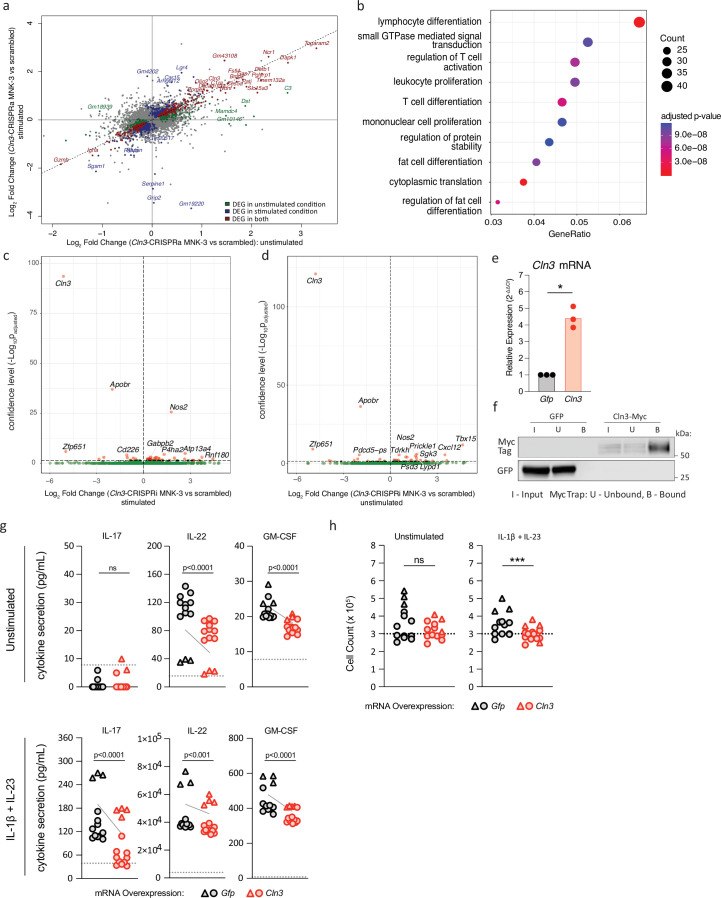
Additional results on the role of CLN3 in ILC3 inflammatory function. **a**, Comparison of differentially expressed genes in IL-23/IL-1β-stimulated vs unstimulated *Cln3*-CRISPRa cells (relative to scrambled gRNA controls). **b**, GO term enrichment analysis for genes differentially expressed upon *Cln3-*CRISPRa stimulation. **c**, Differential expression of genes in IL-23/IL-1β-stimulated *Cln3*-CRISPRi MNK-3 cells vs. scrambled gRNA controls. Red, differentially expressed genes (DESeq2 adjusted *P*-value < 0.05); green, all other genes. **d**, Differential expression of genes in unstimulated *Cln3*-CRISPRi MNK-3 cells vs. scrambled gRNA controls. Red, differentially expressed genes (DESeq2 adjusted *P*-value < 0.05); green, all other genes. **e**, *Cln3* expression in MNK-3 cells electroporated with *Cln3*-myc mRNA or GFP mRNA. Transcript abundance was quantified by qPCR, normalized to *Hprt*, and expressed relative to the GFP mRNA control. Each point represents an independent experiment. Statistical significance was assessed using a paired *t*-test, *P* < 0.05 (*). **f**, Verification of CLN3-Myc protein expression and Myc tag-dependent pulldown. MNK-3 cells were lysed, subjected to immunoprecipitation using Myc-Trap agarose, and resolved by reducing SDS-PAGE. I, input lysate; U, unbound fraction; B, bead-bound fraction. Immunoblotting with anti-Myc tag antibodies detected a ~ 65–80 kDa CLN3-Myc species selectively enriched in the bound fraction. The uncropped Western blot is shown in Supplementary Fig. 2. **g**, Cytokine secretion upon *Cln3* overexpression across independent experiments. MNK-3 cells were electroporated with GFP mRNA (black) or *Cln3*-*Myc* mRNA (red) and cultured for 24 h under unstimulated (top row) or IL-1β + IL-23–stimulated (bottom row) conditions. Cytokine concentrations (IL-17, IL-22, GM-CSF) in culture supernatants were quantified by ELISA. Each symbol represents a biological replicate from two independent experiments (triangles vs. circles). The dotted horizontal line indicates the lower limit of quantification for each assay. Statistical significance was assessed using a linear mixed-effects model with experiment as a random effect and transfection as a fixed effect (*n* = 13–14 per condition). Solid grey lines indicate group means, with dotted grey bands indicating 95% confidence intervals of the fixed-effect. **h**, Cell numbers upon *Cln3* overexpression with and without inflammatory stimulation. MNK-3 cells were electroporated with GFP mRNA (black) or Cln3-myc mRNA (red) and cultured for 24 hr in unstimulated (left) or IL-1β + IL-23–stimulated (right) media. Each symbol represents a biological replicate from two independent experiments (triangles vs. circles). Lines connect the experiment-specific means. Viable cell numbers were quantified by trypan blue exclusion. The dotted line indicates the number of cells seeded at 0 h. Statistical significance was assessed using a linear mixed-effects model with experiment as a random effect and transfection as a fixed effect (*n* = 13–14 per condition). ns, not significant.

To interrogate the transcriptional consequences of stimulation-induced *Cln3* repression, we used CRISPR activation (CRISPRa) based on dCas9–VP64 and MS2-p65–HSF1 to prevent *Cln3* downregulation in MNK-3 cells upon stimulation. CRISPRa targeted to the *Cln3* promoter produced an approximately threefold increase in *Cln3* expression in stimulated MNK-3 cells and an ~2.1-fold increase in the baseline condition (Fig. 6b, left). Notably, *Apobr* expression was also increased in both basal and stimulated conditions (Fig. 6b, right), potentially reflecting local effects of CRISPRa targeting but consistent with the coordinated regulation observed at this locus (Fig. 6b). Bulk RNA-seq analysis revealed widespread transcriptional changes following *Cln3*-CRISPRa, with 519 differentially expressed genes in unstimulated cells and 722 in stimulated cells relative to scrambled guide RNA (gRNA) controls (DESeq2 adjusted *P* < 0.05) (Fig. 6c, Extended Data Fig. 8a and Supplementary Table 13; Data S10 at OSF^15^). These genes were enriched for pathways involved in lymphocyte differentiation, activation and proliferation and included *Il23r*, *Cd74* and *Fas* (upregulated) and inflammatory serine protease genes *Gzmb* and *Gzmc* (downregulated) (Fig. 6d and Extended Data Fig. 8b). More than half the genes differentially expressed in stimulated *Cln3-*CRISPRa cells overlapped with genes altered by IL-23/IL-1β or TL1A stimulation in *Cln3*-unperturbed cells^64^ (Fig. 6e), suggesting that sustained *Cln3* expression counteracts canonical activation-associated transcriptional programs. By contrast, CRISPR interference (CRISPRi; dCas9–KRAB)-mediated knockdown of *Cln3* resulted in few transcriptional changes beyond *Cln3* and *Apobr* themselves (Extended Data Fig. 8c,d and Supplementary Table 13; Data S11 at OSF^15^). The observed changes included upregulation of *Nos2*, which encodes a protein previously implicated in limitation of ILC3-driven intestinal inflammation^63^.

CLN3 is a lysosomal and endosomal protein with established roles in vesicular trafficking, lysosomal homeostasis and protein turnover^69–71^, processes that are central to cytokine storage and secretion. We therefore assessed whether CLN3 overexpression in MNK-3 cells modulated cytokine secretion under basal and inflammatory conditions. As expected, MNK-3 cells constitutively secreted IL-22 and GM-CSF, with further induction of these cytokines upon stimulation, whereas IL-17 production was restricted to stimulated conditions (Fig. 6f, Extended Data Fig. 8g and Supplementary Table 14). CLN3 overexpression, as confirmed by reverse transcription quantitative PCR (RT–qPCR) and immunoblotting (Extended Data Fig. 8e,f), significantly reduced secretion of IL-17, IL-22, and GM-CSF by stimulated MNK-3 cells, with basal IL-22 and GM-CSF secretion also reduced in the absence of stimulation (Fig. 6f and Extended Data Fig. 8g). Viable cell numbers were quantified at the end of cytokine secretion assays and showed no difference under basal conditions, with a modest reduction in *Cln3*-overexpressing cells following stimulation (Extended Data Fig. 8h and Supplementary Table 15).

Collectively, these results highlight CLN3 and the broader *CLN3/APOBR* locus as regulators of ILC3 inflammatory output.

### multiCOGS prioritizes candidate effector genes for six immune diseases with potential roles in ILC3 inflammatory function

**Fig. 7:**
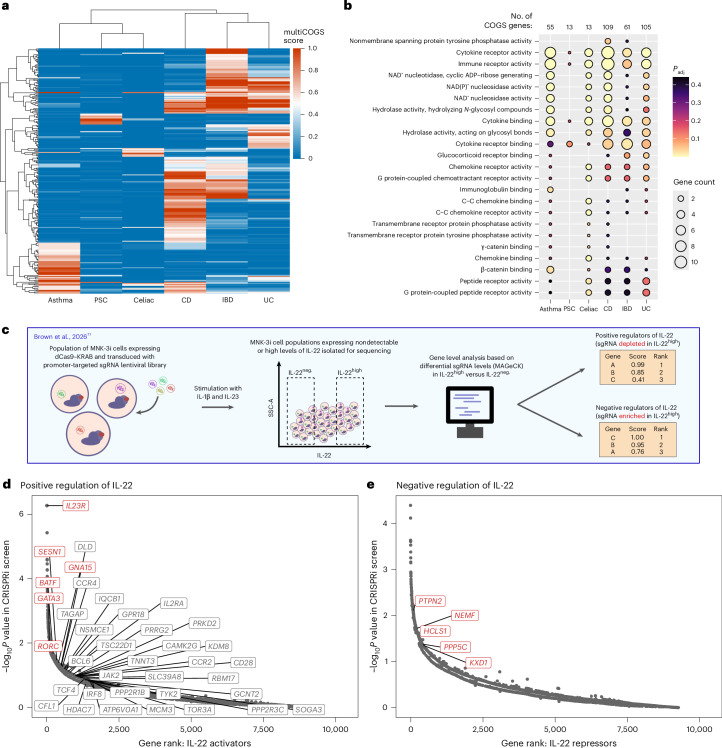
A compendium of prioritized genes in ILC3s for six immune-related diseases. **a**, multiCOGS results across asthma, primary sclerosing cholangitis, celiac disease, Crohn’s disease, IBD and ulcerative colitis in ILC3 cells. Rows represent each gene that scored at least 0.5 in one of the traits. Colors show the multiCOGS score in each trait. Clustering on genes (rows) and traits per cell type (columns) was based on Euclidean distance. **b**, Significant hallmark pathways identified in at least one of the traits in ILC3 cells by gene ontology term analysis (hypergeometric overrepresentation test, with *P* values adjusted for multiple testing via the Benjamini–Hochberg method). **c**, Schematic of the MNK-3i CRISPRi screen for detection of genes involved in regulation of IL-22 signaling^76^. **d**, MultiCOGS genes for all six traits visualized among the CRISPRi results, ranked by evidence of positive IL-22 regulation in MNK-3i cells. The gene rankings and gene-level robust rank aggregation analysis (RRA) *P* values were directly obtained from ref. ^76^. The multiCOGS genes with GSEA adjusted *P* < 0.05 in the screen are labeled in red (GSEA *P* values were adjusted for multiple testing via permutation-derived false discovery rate). multiCOGS genes driving GSEA signal (‘leading edge’) are labeled in gray. **e**, As in **d**, but for genes ranked by score for negative IL-22 regulation in the MNK-3i screen. Red labels indicate multiCOGS genes significant in the screen at GSEA adjusted *P* < 0.05. As GSEA did not identify a significant enrichment of multiCOGS-prioritized genes among IL-22 repressors, the leading edge genes are not labeled. CD, Crohn’s disease; PSC, primary sclerosing cholangitis; UC, ulcerative colitis.

We extended multiCOGS analysis beyond Crohn’s disease to five other immune GWAS datasets with available summary statistics that showed enrichment at active ILC3 and CD4^+^ T cell PIRs in the RELI analysis, including adult-onset asthma^72^, IBD^40^, ulcerative colitis^40^, primary sclerosing cholangitis^73^ and celiac disease^74^ (Supplementary Tables 16–18). Across the six traits and two cell types, we detected a total of 332 prioritized disease candidate genes (multiCOGS score > 0.5), of which 251 were prioritized in ILC3 cells (Fig. 7a) and 266 in CD4^+^ T cells (Supplementary Table 17). As expected from their shared etiology, the three traits relating to Crohn’s disease, ulcerative colitis and IBD clustered together with respect to gene-level multiCOGS scores, whereas asthma formed an outgroup (Fig. 7a).

A total of 66 candidate genes were prioritized in ILC3s only and 81 in CD4^+^ T cells only (Supplementary Table 17). Notable ILC3-specific candidate genes included several cytokines and receptors involved in immune response, such as *CCR2* (celiac disease) and *BCL6* and *IL17A* (asthma), as well as IL-18 receptor gene *IL18R1* (Crohn’s disease, celiac disease and asthma). We also noted ILC3-specific prioritization for Crohn’s disease and asthma of *BTN3A1* and *BTN3A2*, which encode transmembrane butyrophilins involved in recognition of microbial antigens^75^. Finally, *CLN3* was also prioritized for the broader IBD trait (multiCOGS score 0.538) in addition to Crohn’s disease, also selectively in ILC3s.

Pathway analysis of the prioritized genes across all traits revealed shared enrichment for inflammatory gene ontology terms, including cytokine binding and immune receptor activity (Fig. 7b and Supplementary Table 19). To further investigate the roles of these genes in ILC3 inflammatory function, we analyzed a recent CRISPRi screen for regulators of IL-22 expression in IL-23/IL-1β-stimulated MNK-3 cells^76^ (Fig. 7c). We found that six multiCOGS-prioritized candidate genes encoded positive regulators of IL-22 production, and five encoded negative regulators (highlighted in red in Fig. 7d,e) (Supplementary Table 20). Genes encoding positive regulators included established IBD-related IL-22 activators *IL23R*, *BATF* and *RORC*, as well as asthma-associated genes *GNA15*, *SESN1* and *GATA3*. Genes encoding negative regulators included *PTPN2*, *NEMF*, *HCLS1*, *PPP5C* and *KXD1*, with previous evidence for IL-22 repression by *PTPN2* through STAT3 dephosphorylation^77^. Overall, multiCOGS-prioritized genes (labeled in Fig. 7d) were significantly enriched among positive regulators of IL-22 production (gene set enrichment analysis *P* = 3.64 × 10^−2^; Supplementary Table 21), further implicating dysregulated ILC3 activation as a mechanism linking these genes to immune-related disease risk.

In summary, this analysis expands the compendium of prioritized GWAS gene candidates with potential roles in ILC3s to six immune-related disease traits and demonstrates the potential role of many prioritized genes in ILC3 inflammatory function.

## Discussion

Here we report high-resolution promoter interaction profiling in primary human ILC3s, revealing tens of thousands of promoter-anchored contacts, both shared and specific relative to CD4^+^ T helper cells. We found that ILC3 PIRs were enriched for GWAS variants associated with multiple immune diseases, enabling cell-type-resolved gene prioritization based on these data. Using multiCOGS across six immune-related diseases to prioritize risk genes with potential roles in ILC3s, we produced a compendium of 251 genes, including both known and potentially new candidates. The technical challenges and methodological advances of our GWAS gene prioritization framework and a comparison of complementary approaches are examined in the Supplementary Note.

Integration with a CRISPRi screen for genes affecting ILC3 inflammatory response provided an indication of the potential role of these genes in ILC3 biology. This included 11 prioritized genes that were detected as putative IL-22 activators and repressors in the CRISPRi screen^76^. However, further targeted experiments are needed to gain a deeper understanding of the functional role of the prioritized genes in ILC3 biology and their contribution to immune disease risk.

The *CLN3* gene, prioritized in our analysis for Crohn’s disease risk in ILC3s but not in CD4^+^ T cells, underlies the majority of cases of neurodegenerative disorder Batten disease. Although immune features have been reported in Batten disease and other lysosomal disorders^78,79^, the function of CLN3 in the immune system has remained poorly understood. We found that in an ILC3-like mouse cell line, *Cln3* expression was downregulated upon cytokine stimulation of mouse ILC3s, and that *Cln3* overexpression but not knockdown affected stimulation-induced transcriptional programs and cytokine production. These results are supported by the established roles of CLN3 in vesicular trafficking^69–71^. Overall, our findings implicate CLN3 and the broader *CLN3/APOBR* locus in regulation of ILC3 inflammatory function and Crohn’s disease risk, raising the possibility that inflammatory processes may contribute to gastrointestinal manifestations observed in *CLN* deficiency^80^. The regulatory complexity of this locus is examined further in the Supplementary Note.

Human ILC3s in our study were derived from tonsillectomy material, but their regulatory elements showed enrichment for variants associated with immunological disorders affecting a broad range of tissues. This is consistent with findings from single-cell genomics suggesting that cell type rather than tissue type is likely to be the driving factor behind variation in chromatin accessibility and gene expression^81,82^. Furthermore, ILC3s from regularly inflamed tonsils have a cytokine profile closer to that of mucosal-resident ILC3 populations than to that of ILC3s from resting lymph nodes or peripheral blood^83^. Focused studies in relevant physiological contexts and disease models are required to further establish the role of ILC3s in mediating the effects of genetic variation. However, such analyses are complicated by the rarity of ILC3s and a lack of robust human cell line models for this cell type, and by the strong influence of organismal and environmental factors on autoimmune disease pathogenesis, which are difficult to reproduce in a laboratory setting either in vitro or in vivo.

In conclusion, our work presents improved methods for profiling and detection of promoter-anchored chromosomal interactions and use of these data to interpret GWAS signals. We used this framework to generate a comprehensive catalog of ILC3 promoter contacts and detect candidate effector genes implicating these cells in immune-related disease risk, providing initial functional validation of the prioritized candidates. These findings advance our understanding of ILC3 biology and the contributions of this rare cell type to disease, as well as demonstrating the broader utility of our approach for study of regulatory architecture and GWAS interpretation in other rare cell populations.

## Methods

### Human ILC3 isolation

Three children requiring tonsillectomy (two male, one female) were recruited to a prospective study at a tertiary academic care center through the division of Pediatric Otolaryngology-Head and Neck Surgery at Cincinnati Children’s Hospital Medical Center. Consent was obtained from parents in the perioperative suite on the day of the procedure with approval of the institutional review board: ‘Tissues for the study of functional genomics and immunity’ (no. 2018-3069) at Cincinnati Children’s Hospital Medical Center. Criteria for enrollment in the study included a history of sleep-disordered breathing or recurrent or chronic tonsillitis requiring removal of the tonsillar tissue. Individuals were excluded from the study if the tonsillar tissue was acutely infected or if anatomic abnormalities were present that required a more detailed pathologic evaluation after the surgical procedure. Samples were labeled with a deidentified barcode and transferred to the research team for further processing.

Next, tonsils were dissociated into a single-cell suspension as previously described^89,90^. Briefly, human tonsil tissue was processed by mincing with scissors, followed by transfer of up to 4 g of tissue to a gentleMACS C tube (Miltenyi Biotec) containing 8 ml of phosphate-buffered saline (PBS) with 0.5 mg ml^−1^ collagenase D and 3,000 U ml^−1^ DNase I, and then dissociated on a GentleMACS Octo Dissociator (Miltenyi Biotec) using ‘program C (Spleen program 2 followed by spleen program 3).’ Tissue homogenates were incubated in a 37 °C water bath for 15 min and then dissociated again using ‘program C’ and transferred through a 100-μm cell strainer into 20 ml RPMI containing 10% human AB serum (Sigma-Aldrich). Next, the cell suspension was overlaid on 10 ml of Ficoll-Paque PLUS (GE Healthcare) and subjected to density-gradient separation via centrifugation for 20 min at 700*g*, 20 °C, slow acceleration and no brake. Leukocytes were collected from the interphase layer and washed with 50 ml of PBS for 6 min at 560*g*, 20 °C.

Single-cell suspensions of tonsil mononuclear cells were subjected to positive selection with anti-human-CD3, anti-human-CD19 and anti-human-CD14 (Miltenyi Biotec) and transferred through LD columns (Miltenyi Biotec) according to the manufacturer’s guidelines (Supplementary Fig. 1). The depleted cell suspension flowthrough was collected into a 15-ml conical tube and then centrifuged for 5 min at 300*g*, 20 °C. Subsequently, cells were labeled with LIVE/DEAD Fixable Near-IR dead cell stain kit (Invitrogen). Next, cells were labeled with a sorting antibody cocktail that contained negative-linage (Lin^−^) CD19-Brilliant Violet (BV)421 (HIB19), CD14-BV421 (63D3) and CD3-BV421 (OKT3), and the following antibodies: CD45-FITC (HI30), CD94-PerCP-Cy5.5 (DX22), CD127-PE-Cy7 (A019D5), cKit-BV510 (104D2) and NKp44-Alexa Fluor (AF)647 (P44-8), all purchased from BioLegend; and CRTH2-PE (301109) from R&D. ILC3 cells were sorted based on CD45^+^Lin^−^CD127^+^CD94^−^CRTH2^−^cKit^+^NKp44^+^ expression, similar to the method used by Bar-Ephraim et al.^83^. Cells were sorted using a FACSAria II (BD Biosciences). Sorted ILC3 cells were washed with PBS for 5 min at 300*g*, 20 °C, then incubated in 100 μl of 2% formaldehyde (in PBS) for 10 min, followed by addition of 0.125 M glycine. Next, cells were centrifuged at 400*g* for 5 min at 4 °C, resuspended with cold PBS and centrifuged again at 400*g* for 5 min at 4 °C; then, the supernatant was discarded, and cells were snap-frozen in liquid nitrogen and stored at −80 °C before PCHi-C analysis.

### Human CD4^+^ T cell isolation

Samples were obtained from two male donors after written informed consent had been obtained under studies ‘A Blueprint of Blood Cells’ (REC reference 12/EE/0040) and ‘Genes and mechanisms in type 1 diabetes in the Cambridge BioResource’ (REC reference 05/Q0106/20), both of which were approved by the NRES Committee East of England – Cambridgeshire and Hertfordshire. Total CD4^+^ lymphocytes were obtained from PBMCs from venous blood by negative selection using an EasySep Human CD4^+^ T Cell Enrichment kit (catalog no. 19052) from STEMCELL Technologies. Purified CD4^+^ T cells were washed with PBS for 5 min at 300*g*, 20 °C, then incubated in 100 μl of 2% formaldehyde (in PBS) for 10 min, followed by addition of 0.125 M glycine. Next, cells were centrifuged at 400*g* for 5 min at 4 °C, resuspended with cold PBS and centrifuged again at 400*g* for 5 min at 4 °C; then, the supernatant was discarded, and cells were snap-frozen in liquid nitrogen and stored at −80 °C before PCHi-C analysis. Two replicates of 1 million and two more replicates of 50,000 cells were used to generate PCHi-C datasets.

### Promoter capture Hi-C

PCHi-C was performed as previously described^12^. Cells were lysed in a lysis buffer (for 30 min on ice) and digested with *DpnII* (NEB) overnight at 37 °C with rotation (200*g*). Restriction overhangs were filled in with Klenow (NEB) using biotin-14-dATP (Jena Bioscience), and ligation was performed in ligation buffer for 4 h at 16 °C (T4 DNA ligase; Life Technologies). After overnight decrosslinking at 65 °C, the ligated DNA was tagmented to produce fragments of 300–700 bp. Ligation products were isolated using MyOne C1 streptavidin beads (Life Technologies), followed by washing with Wash&Binding buffer and nuclease-free water. Isolated Hi-C ligation products on the beads were then used directly for PCR amplification, and the final Hi-C library was purified with AMPure XP beads (Beckman Coulter). PCHi-C was performed using a custom-designed Agilent SureSelect system following the manufacturer’s protocol. The PCHi-C libraries were paired-end sequenced (100 bp) on an Illumina HiSeq 2500 machine at a sequencing depth of ~400 million reads per sample (Supplementary Table 1).

### PCHi-C data preprocessing and detection of significant interactions

Sequencing data from three ILC3 PCHi-C biological replicates were aligned to the hg38 genome assembly using Bowtie2 (ref. ^91^) and quality-controlled using HiCUP^92^. Quality metrics for all generated PCHi-C datasets are reported in Supplementary Table 1. Significant interactions were then detected across replicates using CHiCAGO^14^ as previously described^16^ at single *DpnII* fragment resolution and in bins of fragments approximately 5 kb in length, with the baited promoter fragments left unbinned. Design files for CHiCAGO analysis are available as Data S12 at OSF^15^. Leaving the baited *DpnII* fragment unbinned meant that nearly every baited fragment was occupied by a single protein-coding gene promoter. By contrast, one-third (33%) of baited fragments in the *HindIII*-based Capture Hi-C design (with a median fragment size of 4 kb) contained two or more promoters. Therefore, leaving the baited fragment unbinned substantially improved the resolution and interpretability of analyses such as (multi)COGS.

For CHiCAGO analysis at single-fragment resolution, *P* value weights were estimated following our previously described procedure^16^ (weights rounded to 2 decimal digits: weightAlpha = 24.67, weightBeta = −2.29, weightGamma = −16.05, weightDelta = −6.99); CHiCAGO default *P* value weights were used for the 5-kb analysis. A score cutoff of ≥5 was used for both resolutions. A consensus list of promoter interactions was compiled from nonredundant contacts detected at fragment and 5-kb resolutions, with fragment-level contacts taking priority over 5-kb contacts in cases of overlap.

For comparison of *DpnII*-based ILC3 PCHi-C data with *HindIII*-based data from Javierre et al.^17^ reported on GRCh37, the ILC3 data were realigned with HiCUP using the GRCh37 assembly and *HindIII* resolution and reanalyzed with CHiCAGO using the *HindIII* PCHi-C design from the same study.

### Activity-by-captured-contact

For a given gene–enhancer pair, the ABC score^22,93^ is the normalized product of enhancer activity (proxied by levels of chromatin accessibility and relevant histone modifications) and contact (proxied by three-dimensional contact frequency detected from a chromosome conformation capture assay):

$$\textrm{ABC score}_{E,G}=\frac{{A}_{E}\times {C}_{E,G}}{\sum_{e\ {\rm{within}}\ 5\ {\rm{Mb}}\ {\rm{of}}\ G}\,{A}_{e}\times {C}_{e,G}},$$

where *E* and *G* are an enhancer and gene of interest, respectively; *e* denotes enhancers in the gene’s 5 Mb neighborhood; *A* is enhancer activity and *C* is enhancer–promoter contact frequency. To adapt ABC for PCHi-C data, we took advantage of the CHiCAGO normalization algorithm and developed an imputation procedure in the normalized counts space based on the inferred decay of interaction read counts with distance. As we did not expect the frequency of enhancer–promoter contacts to fall below levels expected owing to Brownian collision, for a given pair of fragments involving a baited promoter, we selected the maximum between the CHiCAGO-normalized observed read counts (*N*_obs_) and expected read counts *N*_exp_ estimated as:

$${N}_{\exp }={B}_{{\rm{mean}}}/({s}_{{\rm{i}}}\times {s}_{{\rm{j}}}),$$

where *B*_mean_ is the CHiCAGO-estimated Brownian noise level, and *s*_i_ and *s*_j_ are the bait- and other end-specific scaling factors. For promoters that could not be baited in the Capture Hi-C design and those that were filtered out owing to failing quality control, we estimated the expected normalized read count directly from the interaction distance *d*, using the distance function f(*d*) fitted by CHiCAGO. Given the very large values of f(*d*) for very-short-range interactions (<1.5 kb), in the imputation procedure we capped them at the value of f(*d*) for *d* = 1.5 kb (median fragment length) for candidate enhancers that were less than 1 fragment away from the bait. Please refer to Additional File 1 in the publication presenting the CHiCAGO pipeline^14^ for the formal definition of these parameters and their estimation procedures.

The imputed normalized read counts were used as contact data in the ABC pipeline, and public H3K27ac data and ATAC using sequencing (ATAC-seq) data for ILC3s, processed as described above, were used to compute activity. To validate the ABCC approach, we used high-throughput CRISPRi-FlowFISH data from Fulco et al.^22^, which showed the impact of perturbing ~3,500 enhancer elements on the expression of 30 genes in K562 cells. As PCHi-C data for K562 cells were not available, we used our previously published PCHi-C dataset in a related primary cell type, erythroblasts^17^, to generate ABC scores based on these data and the ATAC-seq and H3K27ac chromatin immunoprecipitation followed by sequencing (ChIP–seq) datasets for K562 cells from Fulco et al. In comparison with the original ABC scores from Fulco et al., which were based on pooling of conventional Hi-C data from multiple cell types, our approach showed higher precision (69.1% versus 58.3%) at the same level of recall (58.3%) of CRISPRi-FlowFISH-validated enhancer–promoter pairs (Extended Data Fig. 2). To select the ABCC score cutoff, we optimized the Pearson correlation between the per-gene ABCC numerator and gene expression (*R*_ABC-GE_), using an approach inspired by Xu et al.^94^ We used a single ABCC score cutoff of 0.023 for all analyzed cell types, as this was close to both the maximum *R*_ABC-GE_ in each cell type and the cutoff of 0.02 that yielded an optimal precision–recall of CRISPRi-FlowFISH-validated enhancer–promoter pairs in K562 cells.

### RNA-seq data analysis

Human ILC3 RNA-seq data^21^ were downloaded from the Gene Expression Omnibus (GEO; accession GSE130775). Salmon^95^ was used to map reads to transcripts on the hg38 and mm10 genome assemblies for human and mouse RNA-seq data, respectively. The transcript counts were imported into DESeq2 objects and collapsed to gene counts using Tximport^96^. Differential analysis was performed using DESeq2 (ref. ^88^). CRISPRi and CRISPRa data were analyzed separately, with four conditions (baseline versus stimulated; CRISPR-targeted versus scrambled control) included in each analysis and specific comparisons queried using contrasts. Low-count genes were prefiltered before the analysis was run. The following parameters were used to report significantly differentially expressed genes: alpha = 0.05 and adjusted *P* < 0.05. For the TL1A stimulation experiment, gene count matrices from ref. ^64^ were downloaded from GEO (accession GSE120723) and reanalyzed using DESeq2.

### RELI analysis

RELI determines significantly enriched overlaps between selected genomic loci (here PIRs intersecting open chromatin or H3K27ac signals in ILC3s based on public data) and trait-associated genetic variants. This is done by comparing the observed overlaps with a null distribution of artificially created variant sets with LD characteristics similar to those of the trait-associated variants^28^. A practical advantage of RELI over the commonly used stratified LD score regression^97^ is that it does not require summary statistics data and can be performed on sets of significant single-nucleotide polymorphisms (SNPs) reported in the GWAS Catalog^29^.

RELI^28^ (v.0.1.1a) was used to find enrichment of genetic variants in PIRs that were accessible and/or marked with activating epigenetic markers (H3K27ac and ATAC-seq). In brief, RELI tests genomic features such as ATAC-seq or ChIP–seq peaks or PCHi-C PIRs for statistically significant overlaps with known disease risk variants identified in GWAS. Risk variants are expanded to LD blocks with variants that have *r*^2^ value ≥ 0.8. LD blocks are then intersected with the genetic feature BED files. A null distribution is generated using randomly shuffled LD blocks (*n* = 1,000) and intersection with the feature files. A *P* value is generated by comparing the observed number of intersections in the test to the null distribution.

Promoter-interacting regulatory elements were determined as input for RELI as follows. The PIR sets were the union of PCHi-C interactions (CHiCAGO score ≥ 5, binned to 5-kb or *DpnII* fragment-level resolution) and ABC enhancers, excluding any *trans*-chromosomal interactions. Regulatory elements were then defined as the union of peaks of open chromatin and H3K27ac in ILC3 and CD4^+^ T cells (using ATAC-seq and ChIP–seq data as above). The true intersection between these regulatory elements and PIRs in each cell type was then determined using pybedtools intersect. The coordinates for these regions were lifted over from hg38 to hg19 using UCSC liftOver (v.377), then sorted and merged for use with RELI. RELI^28^ was run against 763 traits of individuals of European ancestry in the GWAS Catalog v.1.0.2. The traits were filtered to those with ≥10 independent risk loci, resulting in a final set of 495 traits in the results. Results with Bonferroni-adjusted *P* < 0.05 were defined as significant. For depiction of RELI results, we labeled only significant traits with total number of risk loci per trait greater than 10.

### Summary-level GWAS imputation and multivariate fine-mapping

We downloaded LD block data for EUR from lddetect^98^ (https://bitbucket.org/nygcresearch/ldetect-data/src/master/) and converted it to hg38 using liftOver^99^. We generated LD matrices based on samples from individuals of European ancestry from phased 1000 Genomes Phase 3 data^100^ downloaded from https://mathgen.stats.ox.ac.uk/impute/1000GP_Phase3.html.

We first used the LD matrices to impute the summary statistics data within blocks using a published method^36^. In brief, given a vector of observed *z*-scores, an estimate of the correlation between observed variations *S*, and an estimate of the correlation between an unobserved variant *u* and observed markers *s*, we imputed the *z*-score at the unobserved variant as:

$${z}_{u}={s}^{{\rm{T}}}{S}^{-1}z.$$

To ensure *S* was invertible, we pruned observed variants to ensure there were no pairs of variants in high LD (*r*^2^ > 0.8).

For blocks with appreciable association signals (minimum *P* < 10^−6^), we used the susieR package^37,38^ to fine-map the data. We defined ‘detected signals’ as those for which SuSiE could calculate a 95% credible set and used the posterior inclusion probabilities (PIP) for each SNP for each signal thus detected as input for multiCOGS. For the remaining blocks, or for cases in which susieR failed to find any signals meeting our criteria, we fine-mapped using the single causal variable approach, as previously described^17,35^, and used the posterior probabilities of association as input for multiCOGS.

### multiCOGS

We modified the standard COGS algorithm^17,35^ to account for the inclusion of multiple association signals in a region (multiCOGS). Whereas in standard COGS, fragment-level scores are calculated by summing variant-level posterior inclusion probabilities within a given fragment and LD block, multiCOGS considers each credible set within each LD block and forms an overall gene score as the probability that at least one of the multiple fine-mapped signals is linked, through PCHi-C, to the gene of interest:

$${\rm{multiCOGS}\, \rm{score}}_{\rm{gene}}=1-\prod (1-{\rm{score}}_{{\rm{gene},\ \rm{LD}\ \rm{block}},\ {\rm{credible}\ \rm{set}}}).$$

As input for multiCOGS, we used imputed and fine-mapped summary-level GWAS data alongside CHiCAGO- and ABCC-processed PCHi-C results (thresholded at CHiCAGO score ≥ 5 and ABCC scores ≥ 0.023). In addition, for each transcription start site annotated in Ensembl GRCh38 release 88 (Havana and Ensembl/Havana merge), regardless of whether it was included in the PCHi-C capture design, we defined virtual promoter regions as windows of ±5 *DpnII* fragments surrounding the fragment containing the transcription start site. Finally, we provided the locations of protein-coding SNPs identified using VEP^101^ v.99.2 (https://github.com/Ensembl/ensembl-vep). The results for each protein-coding gene were linked across datasets using Ensembl gene IDs as primary identifiers. We removed the major histocompatibility complex locus (GRCh38 6:28510120-33480577) before running multiCOGS.

To determine the contributions of the four categories of genomic loci underlying the prioritized genes (PCHi-C PIRs, ABCC enhancers, virtual promoter regions and coding SNPs), we also ran multiCOGS on each category separately by specifying the feature.names argument in the compute_cogs function.

### Cell culture

Mouse MNK-3 cells^61^ (Sigma-Aldrich, SCC628) and derived lines were cultured in DMEM with glucose/pyruvate/l-glutamine supplemented with 10% fetal bovine serum, 1× penicillin–streptomycin, 10 ng ml^−1^ mouse recombinant IL-2 and IL-7 (R&D Systems), and 50 µM 2-mercaptoethanol. Media for CRISPRi MNK-3 (MNK-3i) cells contained 10 µg ml^−1^ blasticidin S, and media for CRISPRa MNK-3 (MNK-3a) cells contained 10 µg ml^−1^ blasticidin S and 1,250 µg ml^−1^ hygromycin B. MNK-3i/a cells with single-guide RNA (sgRNA) also received 2 µg ml^−1^ puromycin. MNK-3 activation was induced with 10 ng ml^−1^ IL-1β and 10 ng ml^−1^ IL-23 (R&D Systems).

### CRISPRa and CRISPRi

MNK-3i cells were generated as previously described^102^ from parental MNK-3 cells (Sigma-Aldrich, SCC628). In brief, MNK-3 cells were transduced with lentivirus containing pLenti CMV rtTA3 Blast (Addgene 26429), selected with blasticidin S, and then infected with TRE3G-dCas9-KRAB-P2A-mCherry lentivirus^102^. Following incubation with doxycycline, mCherry-positive cells were subcloned, and western blot analysis was performed to confirm robust expression of doxycycline-inducible dCas9–KRAB. MNK-3a cells were lentivirally engineered from MNK-3 to constitutively express the dCas9–VP64 fusion gene (Addgene 61425) and the MS2-p65–HSF1 transactivator complex (Addgene 89308), selected with blasticidin S and hygromycin B, and subcloned. All cells were tested for mycoplasma.

Sequences for Cln3-targeting and scrambled gRNAs were based on published sgRNA libraries for MNK-3i^103^ and MNK-3a^104^ cells and are listed in Supplementary Table 22 alongside RT–qPCR primer sequences. sgRNA sequences and their reverse complements were synthesized by Sigma, annealed, and cloned into a lenti sgRNA(MS2)_puro optimized backbone (Addgene 73797) for MNK-3a or sgOpti (Addgene 85681) for MNK-3i cells using Esp3I digestion as previously described^105^. sgRNA plasmid integration was confirmed by Sanger sequencing (Ohio State Comprehensive Cancer Center Genomics Core). Lentiviral plasmids pMD2.G (Addgene 12259) and psPAX2 (Addgene 12260) were transfected with the sgRNA plasmid into HEK293T cells using Mirus TransIT-293T transfection reagent. Lentivirus media were harvested and filtered 48–72 h posttransfection. Puromycin selection began 36 h after lentiviral guide transduction into MNK-3i/a cells in the presence of polybrene. Bulk transduced populations were used for experiments and maintained in selection antibiotics. RT–qPCR was performed to confirm repression (in MNK-3i lines after 48 h doxycycline incubation) or overexpression (in MNK-3a cells) of target genes relative to *Actb* and the respective scramble control (TRIzol RNA isolation; Verso cDNA synthesis).

To induce CRISPRi guide expression, MNK-3i cells stably expressing Cln3-targeting and scrambled gRNAs were incubated with 2 µg ml^−1^ doxycycline for 48 h. To confirm stimulation, cells were harvested 21 h after cytokine stimulation and stained for intracellular IL-17F and IL-22 (eBioscience IL-22 clone 1H8PWSR and IL-17F clone eBio18F10; BD Life Sciences Cytofix/Cytoperm kit). Expression of IL-17F and IL-22 was assessed on a FACSymphony (BD Life Sciences) and compared against the respective scrambled control.

### Design and generation of in vitro-transcribed mRNA

The protein-coding sequence of mouse *Cln3* was based on the longest annotated transcript (NM_001146311.3/ENSMUST00000084589.11). A Myc epitope tag was inserted near the amino terminus, between amino acid residues 3 and 4, within a predicted disordered and cytoplasmic region of the protein. The resulting coding sequence was synthesized and used for in vitro transcription by ApexBio.

In vitro-transcribed mRNA was generated with a cap 1 structure and incorporated *N*_1_-methylpseudouridine. Transcripts contained a poly(A) tail and were supplied in RNase-free sodium citrate buffer (pH 6.4) at a concentration of 1 mg ml^−1^. Control mRNA encoding GFP was generated using the same chemistry.

### mRNA electroporation and cytokine stimulation

MNK-3 cells were electroporated with mRNA using an ATx electroporation system (MaxCyte); 1.0 × 10^7^ cells were electroporated in a 100-µl reaction containing 20 µg of GFP or *Myc*-tagged *Cln3* mRNA (2 µg per 10^6^ cells) using the ‘Optimization 8’ program. Following electroporation, cells were rested for 15 min at 37 °C and then incubated for 15 min at 37 °C in prewarmed medium supplemented with 10 µg ml^−1^ DNase I (Thermo Fisher Scientific), 5 mM MgCl_2_ and 1 mM CaCl_2_ before being transferred to complete MNK-3 culture medium.

At 24 h postelectroporation, cells were seeded at 3.0 × 10^5^ cells per well in 24-well plates. Transfected cells were cultured for an additional 24 h in the presence or absence of recombinant mouse 10 ng ml^−1^ IL-1β and 10 ng ml^−1^ IL-23 (R&D Systems). At 48 h postelectroporation, supernatants were collected, clarified by centrifugation and stored at −20 °C. Viable cell numbers were determined by trypan blue exclusion.

### Enzyme-linked immunosorbent assay

Cytokines in cell culture supernatants were quantified by enzyme-linked immunosorbent assay using DuoSet kits for mouse IL-17, IL-22 and GM-CSF (R&D Systems) according to the manufacturer’s instructions. When necessary, samples were diluted to fall within the dynamic range of the standard curve. Absorbance was measured at 450 nm with wavelength correction at 560 nm using a GloMax Discover microplate reader (Promega). Cytokine concentrations were determined by interpolation from standard curves using a four-parameter logistic fit.

Data were analyzed using GraphPad Prism. Statistical significance was assessed using unpaired Welch’s *t*-tests (single experiment) or linear mixed-effects models with genotype as a fixed effect and experiment as a random effect (multiple experiments).

### Immunoprecipitation and immunoblotting

MNK-3 cells were electroporated with GFP or Myc-tagged *Cln3* mRNA as described above and harvested 24 h later. Cells were lysed in a nondenaturing buffer containing 50 mM Tris-HCl, 150 mM NaCl, 1 mM EDTA, 1% *n*-dodecyl-β-d-maltoside, 10% glycerol and protease phosphatase inhibitors (Thermo Fisher Scientific). Lysates were clarified by centrifugation at 4 °C.

Myc-tagged proteins were enriched by incubation of clarified lysates with Myc-Trap agarose beads (ChromoTek) for 1 h at 4 °C with rotation. Beads were washed in a buffer containing 0.05% *n*-dodecyl-β-d-maltoside, and bound proteins were recovered for analysis. Input, unbound and bound fractions were quantified by BCA assay (Thermo Fisher Scientific), denatured in LDS sample buffer with reducing agent and resolved by SDS–PAGE on 4–12% Bis-Tris gels (Thermo Fisher Scientific). Proteins were transferred to polyvinylidene fluoride membranes, stained with Revert 700 Total Protein Stain (LI-COR) and imaged before immunoblotting.

Membranes were blocked and probed with antibodies against the Myc tag (Cell Signaling Technology 2278, 1:1,000) or GFP (Invitrogen A-11122, 1:2,000). Fluorescent secondary antibodies were used at 1:10,000, and blots were imaged using an Odyssey DLx Imaging System (LI-COR).

### Querying a CRISPRi screen for regulators of ILC3 inflammatory response for multiCOGS-prioritized genes

We downloaded Supplementary Table 5 from Brown et al.^76^ and filtered it to include 8,149 genes that had been profiled in the multiCOGS experiment, based on an identical gene name between the mouse and human data. These genes were ranked based on their MAGeCK^106^ score for positive or negative regulation of IL-22 production. We then ran gene set enrichment analysis against each of these rankings for the 142 multiCOGS genes for inflammatory traits, using the ‘pathway’ function in MAGeCK (v.0.5.9.5) with 100,000 permutations. We considered significant CRISPRi genes to be those with adjusted *P* < 0.05 in the gene-level robust rank aggregation analysis.

### Reporting summary

Further information on research design is available in the Nature Portfolio Reporting Summary linked to this article.

## Online content

Any methods, additional references, Nature Portfolio reporting summaries, source data, extended data, supplementary information, acknowledgements, peer review information; details of author contributions and competing interests; and statements of data and code availability are available at 10.1038/s41588-026-02681-0.

## Supplementary information

Supplementary Information Supplementary Note and Figs. 1 and 2.

Reporting Summary

Supplementary Tables 1–22 Supplementary Tables 1–22.

## Data Availability

Raw PCHi-C data generated in this study for ILC3s have been deposited at GEO under accession code GSE216267. Processed R data files containing CHiCAGO scores at fragment-level and 5-kb binned resolution can be found in the same repository. PCHi-C data for CD4^+^ T cells were deposited to the European Genome-Phenome Archive under managed access in accordance with the conditions of donor consent, under accession code EGAS50000001316. Raw RNA-seq reads and counts for the *Cln3*-CRISPRi and *Cln3*-CRISPRa experiments in MNK-3 cells have been deposited at GEO under accession code GSE313942. Large data files S1–S12, including significant CHiCAGO interactions at fragment-level and 5-kb resolution in ILC3 and CD4^+^ T cells, ABCC pairs in both cell types, and DESeq2 objects for the *Cln3*-CRISPRi and *Cln3*-CRISPRa experiments, were deposited at OSF^15^ (https://osf.io/aq9fb).

## Code availability

All code repositories generated in this study, including ABCC, RELI, multiCOGS, fine-mapping and imputation, and custom analysis scripts, are available via GitHub at https://github.com/topics/malysheva-et-al, and their snapshots at the time of acceptance of this paper have been archived at Zenodo^107–112^.
