# Supplementary material for: High-resolution promoter interaction analysis implicates genes involved in activation of type 3 innate lymphoid cells in immune disease risk
Source: Nat Genet. 2026 Aug 4;58(8):1953–66. doi: 10.1038/s41588-026-02681-0 (PMC13447110; doi:10.1038/s41588-026-02681-0)
Supplement: Supplementary file 1 [file 41588_2026_2681_MOESM1_ESM.pdf]

nature genetics

Supplementary information https://doi.org/10.1038/s41588-026-02681-0

# High-resolution promoter interaction analysis implicates genes involved in activation of type 3 innate lymphoid cells in immune disease risk

In the format provided by the authors and unedited

**Malysheva, Ray-Jones, Lakes et al.**

# High-resolution promoter interaction analysis implicates genes involved in the activation of type 3 innate lymphoid cells in autoimmune disease risk

## Supplementary Information:

- **Supplementary Note**, including Supplementary Results, Methods and Discussion
- **Supplementary Figure 1**

## Supplementary Note

## Supplementary Results

### ABCC validation using public data

To validate the ability of the ABCC algorithm to detect functional enhancer-promoter pairs, we took advantage of CRISPR interference (CRISPRi) enhancer perturbation data in K562 cells, which was generated to validate the original ABC approach[1]. As inputs for ABCC, we used public epigenetic annotations in K562 cells and our previously generated high-coverage PCHi-C data in their physiological counterparts, erythroblasts[2]. These analyses demonstrated the power of ABCC to predict functional enhancer-promoter links from lineage-relevant PCHi-C and chromatin readouts (**Extended Data Fig. 2c**). In contrast, using PCHi-C data from lymphoid cells at an equivalent coverage mildly reduced ABCC performance (**Extended Data Fig. 2c)**. The strong performance of the ABC algorithm based on pooled Hi-C data (**Extended Data Fig. 2c)** was expected, given the high representation of deeply sequenced data in K562 cells in the pool. In addition, joint clustering of the ABCC profiles generated for four primary blood cell types successfully reconstructed the lineage relationships between them (**Extended Data Fig. 2d**). These results highlighted the potential of ABCC to infer lineage-specific *cis*-regulatory architecture. In comparison with CHiCAGO, ABCC generally detected shorter-range promoter interactions, which was expected due to its reliance on raw contact frequencies (**Extended Data Fig. 2e**). Both ABCC- and CHiCAGO-detected contacts were enriched for markers of accessible (DNase-seq) and/or active (H3K27ac) enhancers, with regions called by both approaches showing the highest enrichment for these marks (**Extended Data Fig. 2f**). Taken together, these results suggest that ABCC and CHiCAGO detect complementary subsets of regulatory promoter contacts.

### Pathway analysis of genes associated with differential promoter contacts between ILC3 and CD4+ T cells

Genes with increased ILC3-specific chromatin contacts were enriched for annotation terms such as “regulation of innate immune response,” including *NFKB1* (NF-κB signaling), *TLR3* (innate immune receptor), and *IFNG* (effector cytokine), and “regulation of immune effector process”, including *IL23R* (controlling ILC3 activation and cytokine production), *IL1R1*, *TNFSF4*, and *SOCS5* (negative feedback on cytokine signalling) (**Fig. 3c; Extended Data Fig. 3a; Supplementary Table 2**). In contrast, genes with CD4+ T cell-specific contacts were involved in “regulation of T cell activation” (e.g. *CD3E, CD86, CTLA4, IL6, FOXN1*) and “negative regulation of the MAPK cascade” (e.g. *DUSP14, DUSP16, PTPN6*) (**Fig. 3c; Extended Data Fig. 3b; Supplementary Table 3**).

We also identified 194 genes with differential contacts between ILC3s and CD4+ T cells, including *BCL2, FYN, CD226* (activating receptor on T and NK/ILC3-like cells), and *CCR7* (guiding ILC3 positioning and migration) (**Fig. 3c; Extended Data Fig. 3c; Supplementary Table 4**). Notably, many genes with ILC3- and/or CD4+ T cell-specific contacts converged on pathways such as TCR signalling and T cell activation (e.g. *IL23R, RORC, NFKB1, CD300A, PIK3R1, ZAP70, CTLA4, CD3E, CD226, ITK, CD28, CCR7*). In contrast, genes with similar contact profiles across both cell types were associated with processes such as histone modification, chromatin remodelling, and lymphocyte proliferation

and differentiation (**Extended Data Fig. 3; Supplementary Table 5**), reflecting their shared functionality in both cell types.

## Pathway analysis of multiCOGS-prioritised CD risk genes

We explored the biological functions of the 109 prioritised CD-risk candidate genes in ILC3s based on their public gene set annotations from MSigDB[3] (**Supplementary Table 9**). Seven Hallmark gene sets were significantly enriched among the candidates, including IL6-JAK-STAT3 signalling, TNFα signalling via NFκB, IL2-STAT5 signalling, inflammatory response, allograft rejection, IFNγβ response, and TGFβ signalling (**Extended Data Fig. 6a**). The enriched Molecular Function GO terms included cytokine receptor activity and NAD+ metabolic activity (**Extended Data Fig. 6b).** The strongest enrichment of cell-type signatures was for tissue-resident immune cells, including gastric and duodenal immune cells, as well as monocytes, dendritic cells, and basophils in the lung (**Extended Data Fig. 6c**). We also noted the signature for ILC progenitor cells in the fetal lung[4], driven by the genes *IL1R1, ICAM1, IFNGR2, PLCG2, CCR6,* and *RORC* (adjusted p = 0.0176), as well as for T cell differentiation and IL-18 signalling, a key cytokine for ILC3 function[5] (**Extended Data Fig. 6c**). The enriched curated WikiPathways[6] highlighted immune-mediated diseases, including rheumatoid arthritis, neuroinflammation, IBD, and bacterial infection (**Extended Data Fig. 6d**). We also noted an enrichment for genes that are differentially expressed in the rectum of patients with CD and ulcerative colitis[7] (UC) (**Extended Data Fig. 6e**; **Supplementary Table 9**).

## Comparison of multiCOGS with standard COGS

We explored more closely how the results of multiCOGS compared with those from our previously published COGS pipeline, which used univariate fine mapping without imputation and was based purely on CHiCAGO results without ABCC (hereafter referred to as "classic COGS"). Classic COGS resulted in substantially smaller prioritised gene sets (55 genes in ILC3 cells and 75 genes in CD4+ T cells with a COGS score > 0.5) (**Supplementary Table 10**). As examples, we note that compelling candidate genes such as *IL12RB2* and *IL15RA* (in ILC3s), *TNFSF15* and *ICAM3* (in CD4s), and *NFKB1*, *BATF*, *ICAM1* and *TNFSF8* (in both cell types) were only prioritised in multiCOGS (**Supplementary Table 10**). Moreover, we discovered that both of the novel aspects of multiCOGS (imputation and multivariate fine mapping) contributed substantially to the increased number of genes prioritised in comparison with classic COGS (**Extended Data Fig. 7a**). For the majority of genes, multiCOGS prioritisation scores were similar or higher than in conventional COGS in both ILC3s and CD4s (**Extended Data Fig. 7b**).

Only five genes prioritised by classic COGS had sub-threshold scores in multiCOGS (*JAK2*, *CREM*, *FRS2*, *IL18R1* and *RP11-894J14.5*; **Extended Data Fig. 7b**). Of these, we were intrigued by the loss of *JAK2* in both cell types, because it is a well-noted candidate gene in IBD, with JAK inhibitors already used to treat ulcerative colitis and CD[8]. The COGS score for *JAK2* was substantially lower across both cell types when genetic imputation and multivariate fine mapping were employed (classic COGS score ~1 in both cell types, multiCOGS score ~0.01 in ILC3s and ~0.03 in CD4s). Upon examining the locus, we discovered that fine mapping with the univariate methodology (Wakefield synthesis[9]) identified the most likely causal variant as rs1887428 (PIP = 0.999) at the *JAK2* promoter. However, summary statistics imputation combined with SuSIE[10] multivariate fine mapping

prioritised the variant rs1327500 (PIP = 0.663) in a region ~20 kb upstream of *JAK2*, without detectable promoter contacts in ILC3 cells or CD4+ T cells (**Extended Data Fig. 7c**). However, considering that both rs1887428 and rs1327500 are known eQTLs for *JAK2* in blood cells[11], *JAK2* remains a strong candidate in this locus by genetic association.

### Inspection of the GWAS locus driving CLN3 prioritisation

*CLN3* was prioritised by multiCOGS based on the overlap of two regions with credible set CD SNPs. The first region is an ILC3-specific *CLN3* PIR located 14.2 kb downstream of the canonical *CLN3* TSS (red band in **Fig. 6a**). The second region lies between exons 10 and 11 of the canonical *CLN3* transcript, adjacent to an annotated internal promoter (first dark blue band in **Fig. 6a**). Unexpectedly, we found that both regions lacked chromatin accessibility and enhancer activity signals in ILC3s, as well as in all other cell types included in the Ensembl Regulatory Build database (**Fig. 6a**). Data from lymphoblastoid cell lines[12] showed enrichment for the H3K36me3 mark, which is typically associated with transcriptional elongation[13] and facultative heterochromatin[14] (**Fig. 6a**). To seek complementary evidence for a regulatory role of this locus, we queried the OpenTargets database[15] for possible colocalisation between the CD risk signal and known *CLN3* expression quantitative trait loci (eQTLs). CD risk GWAS and *CLN3* expression were likely to share a joint causal genetic signal (posterior probability ≥ 0.8, as determined by coloc[16] and reported in OpenTargets) in whole blood[17,18], monocytes[19,20], thyroid[18], small intestine[18], and cerebellum[18]. Notably, the same CD GWAS signals also colocalised with eQTLs for nearby genes, including *APOBR*, which is located ~2 kb downstream of *CLN3* in a divergent orientation, suggesting a complex regulatory architecture at this locus.

## Supplementary Discussion

### The technical challenges and methodological advances of our GWAS gene prioritisation framework

ILC3s are a rare cell type that cannot be easily expanded *in vivo*, which makes their chromosomal interaction profiling challenging. Indeed, this problem precluded ILC3 profiling by standard Hi-C alongside type 2 ILCs in a recent mouse study[21]. Robust Capture Hi-C profiling typically requires even higher cell numbers. Our efficient PCHi-C protocol[22] and the use of a four-cutter enzyme (*DpnII)* have enabled a higher-resolution analysis of human ILC3s in this study, adding these clinically-relevant cells to the ever-expanding array of cell types with available promoter interactome maps, including the 17 abundant blood cell types that we profiled previously using high-coverage PCHi-C at a six-cutter enzyme (*HindIII)* resolution[2]. While emerging technologies provide complementary solutions for the inference of enhancer-promoter relationships, such as through the correlated activities of these elements across cell types or single cells, genetic evidence and high-throughput perturbation screens, 3D genomics-based approaches continue to offer unique advantages by delivering mechanistically-grounded information in high throughput at a reasonable cost and time investment.

Unlike in our previous studies, here we take advantage of two conceptually different computational analysis strategies for detecting promoter contacts from Capture Hi-C data. The first strategy is based on our established CHiCAGO pipeline to detect ‘significant contacts’ – i.e., those whose frequency significantly exceeds the expectation at a given

distance and technical noise levels. The second strategy is based on ABCC, our adaptation of the ABC approach[1,23] to Capture Hi-C data that considers raw contact frequency rather than its statistical significance relative to the distance-dependent background. As expected from this conceptual difference, ABCC prioritises shorter-range contacts compared with CHiCAGO, resulting in the largely non-overlapping sets of identified contacts and GWAS-prioritised genes. However, the longer-range contacts detected using CHiCAGO, which were also enriched for active enhancers, drive the majority of our identified disease associations. From the practical point of view, therefore, these two approaches are largely complementary, and their combined use is warranted. Mechanistically, this suggests that at short linear distances, the background frequencies of promoter-enhancer contacts arising from constrained Brownian motion are sufficient for the functional interactions between these regions. In contrast, at longer ranges, additional factors (e.g., cohesin-mediated loops) are likely required to facilitate the statistically unusual contact frequencies and enable functional interactions.

We find a strong enrichment of CD-associated SNPs within ILC3 PIRs, consistent with recent findings showing that superenhancers specific to ILC3 or Th17 cells, rather than to ILC1 or Th1 cells, preferentially contain CD-associated variants[24]. Using our multiCOGS strategy that integrates GWAS data processed with multivariate statistical fine-mapping with information on enhancer-promoter links from PCHi-C, we prioritise a total of 109 genes in ILC3s, 29 of which are not detected in CD4+ T cells. Notably, the number of multiCOGS-prioritised genes has increased considerably compared with the results obtained with our previously developed COGS pipeline[2,25]. The key improvements of multiCOGS include summary statistics-based imputation and allowing for multiple causal variants per linkage disequilibrium (LD) block. At the molecular level, the increased recall of prioritised genes reflects the fact that the same LD block often contains multiple regulatory elements (including promoter-proximal and distal enhancers). Variants within each of these elements may have largely independent effects from one another[26,27] and from those within protein-coding regions[28]. Furthermore, we identify cases, such as *IKZF1* and *DDC*, where multiple causal variants in the same LD block intersect the regulatory elements of different candidate genes, leading to their joint prioritisation. These results reinforce the notion that the assumption of a single causal variant per LD block used by many established GWAS analysis methods (particularly those based on summary data) is unnecessarily restrictive and may miss key genetic mechanisms underpinning disease processes.

**Complementary gene prioritisation approaches**

While the enrichment of GWAS signals within enhancers was first demonstrated over a decade ago,[29] with the first studies leveraging 3D information for enhancer-gene assignment following shortly thereafter[30–32], the majority of GWAS gene prioritisation studies to date still do not consider 3D chromosomal data[33]. Nonetheless, several computational approaches for variant-to-gene assignment integrating fine-mapped GWAS signals with 3D genomics information and other sources of evidence are now becoming available. For example, FUMA SNP2GENE provides the option to identify candidate genes via enhancer-promoter interactions, but does not integrate fine-mapping SNP probabilities[34]. In addition, the L2G (locus-to-gene) pipeline uses a machine learning algorithm that integrates multiple features, including Capture Hi-C[35]. L2G provides an interpretable output that shows the relative contributions of many factors, including QTL colocalisation, genomic distance, VEP scores[36], and enhancer-promoter interactions, towards an overall gene score per credible set. L2G is

available on the OpenTargets platform[37], but it is not easily adaptable to new functional data. Finally, H-MAGMA incorporates Hi-C-derived chromatin interactions to refine SNP-to-gene assignment for non-coding GWAS variants, but does not integrate them into a probabilistic framework[38]. MultiCOGS complements these efforts by providing an unsupervised and interpretable Bayesian framework based on cell-type-specific, mechanistically-grounded readouts that can be applied to 3D genomic data in cell types relevant to the disease context.

### The regulatory complexity of the CLN3 locus

The region harbouring the fine-mapped CD susceptibility variants in the *CLN3* locus lacks active chromatin signals in ILC3s and other cell types represented in the Ensembl Regulatory Build. This suggests that regulatory activity at this locus may be highly context-specific, potentially emerging only under inflammatory conditions or within discrete cellular states. Supporting this notion, H3K36me3 deposition across this region in lymphoblastoid cell lines was recently proposed as a mark of enhancers that are 'poised' for rapid activation[39]. However, CD-associated variants in this locus may also exert regulatory effects through alternative mechanisms. Several fine-mapped variants in the *CLN3* locus are linked to alternative polyadenylation of the *CLN3* transcript's 3'UTR across multiple tissues[40,41], a mechanism that can influence mRNA stability and translational efficiency and is increasingly recognised as a contributor to complex disease risk[41]. In addition, C*LN3* was reported to undergo splicing-dependent transcriptional activation[42], further expanding the range of potential regulatory mechanisms operating at this locus. The regulatory complexity of the *CLN3* locus is further augmented by its detection as an eQTL for multiple neighbouring genes across diverse cell types. In monocytes, this locus is also an eQTL for the known CD gene *IL27,* with an opposite direction of allelic effect and a lower statistical significance relative to *CLN3* itself[20,43]. Notably, *IL27* is not appreciably expressed in either mouse or human ILC3s. In addition, *CLN3* shares eQTLs with and is divergently expressed from the apolipoprotein B receptor gene *APOBR*. Consistent with this, we show that *Cln3* and *Apobr* are co-regulated upon IL-23/IL-1β stimulation in a mouse ILC3-like cell line. APOBR has a recognised role in lipid uptake in myeloid cells[44], but its function in the lymphoid compartment remains unclear and is likely mechanistically distinct from that of CLN3.

## Supplementary Methods

### RNA isolation and quantitative RT–PCR

Total RNA was isolated from snap-frozen cells using QIAshredder columns and the RNeasy spin-column system (QIAGEN). Complementary DNA (cDNA) was synthesised using the High-Capacity cDNA Reverse Transcription Kit (Thermo Fisher Scientific). Quantitative PCR was performed using TaqMan chemistry with TaqMan Fast Advanced Master Mix (Thermo Fisher Scientific) on a QuantStudio 5 Real-Time PCR System (Thermo Fisher Scientific). Cln3 expression was quantified using the TaqMan Gene Expression Assay Mm00487021_m1 and normalised to the housekeeping gene Hprt using assay Mm03024075_m1. Reactions were performed in technical triplicate. Relative gene expression was calculated using the ΔΔCt method, with MNK-3 cells electroporated with GFP mRNA used as the reference control condition.

## RNA-sequencing

RNA was harvested by spin column (Qiagen RNeasy kit) for polyA-selected 2x150bp bulk RNAseq (Illumina platform, University of Cincinnati Genomics, Epigenomics, and Sequencing Core, Cincinnati, OH, USA). RNA-seq samples were generated in triplicate. Raw paired-end RNA-seq reads were quantified using *kallisto* (v0.48.0) against the mouse reference transcriptome (GENCODE release M32, GRCm39). Transcript indices were first generated with `kallisto index`, and transcript abundances were quantified for each sample using `kallisto quant` with 100 bootstrap replicates. Transcript-level abundance estimates were subsequently summarised to the gene level in R using the *tximport* package (v1.30.0) together with a transcript-to-gene mapping file. Sample metadata, including experimental condition, CRISPR status, and replicate information, were compiled into a metadata table. Gene-level count matrices generated by *tximport* were then used as input for normalisation and differential expression analysis with *DESeq2* (v1.38.0). Sample metadata, including experimental condition, CRISPR status, stimulation, and replicate information, were compiled into a metadata table. Gene-level count matrices were then used for normalisation and differential expression analysis with DESeq2 (v1.38.0). A variance-stabilising transformation (rlog) was applied for visualisation and principal component analysis to identify batch effects. Differential expression analyses were performed using linear models incorporating relevant covariates. For wild-type samples, stimulation status was tested while including CRISPR type as a batch covariate. For CRISPRa and CRISPRi samples, models including interaction terms between CRISPR treatment and stimulation were used to assess treatment-specific effects. Adjusted p-values were calculated using the Benjamini-Hochberg method, and genes with adjusted p-values $< 0.05$ were considered statistically significant.

## Integration of *DpnII*- and *HindIII*-based Promoter Capture Hi-C data

Our previous PCHi-C study in 17 abundant human primary blood cell types, including both lymphoid and myeloid cells[2] was performed using a 6 bp restriction enzyme *HindIII*, unlike the 4-bp cutter enzyme *DpnII* used in the current study. Since restriction fragment size affects the distance distribution of contacts detected in Hi-C-related methods[46–48], direct comparison across these two datasets is challenging. To partially address this issue, we pooled the reads in the *DpnII*-based ILC3 data into genomic windows corresponding to *HindIII* fragments and re-processed the data with HiCUP using the hg19 genome assembly and *HindIII* parameters. We then identified significant interactions using CHiCAGO[49] with the default *HindIII*-based parameters and integrated them with the significant interactions from the Javierre et al. study[2]. To assess the similarity of promoter-interaction patterns in ILC3s with the cell types profiled in Javierre et al., we first ran a joint PCA analysis. We noted that PC1 (accounting for <10% of the variance) clearly segregated the three ILC3 replicates from the remaining cell types, and therefore most likely corresponded to the difference in PCHi-C methods, resolution and sequencing depth. We disregarded PC1 and focused on PC2, PC3, and PC4, accounting for 6.16%, 3.7%, and 3.16% of variance across all tissues, respectively (components beyond PC4 accounted for <3.1% of variance each and were disregarded). For visualisation purposes, we combined these three components using the UMAP non-linear dimensionality reduction algorithm implemented in the umap package in R[50], obtaining the plot shown in **Extended Data Fig. 1a.**

**Alternative promoter analysis**

We used the CHiCAGO results for ILC3 PCHi-C data at 5 kb resolution to profile PIR sharing between alternative promoters. First, we identified a set of genes that had more than one baited promoter, with each promoter having at least one significant interaction with a CHiCAGO score of ≥5 with ≥5 reads. We defined fully shared PIRs as those that interacted with all baited alternative promoters for the same gene, and partially shared PIRs as those that interacted with a subset of alternative promoters for the same gene. We defined distinct PIRs as those that only interacted with a single promoter fragment (CHiCAGO score ≥5). To increase the stringency with which we called PIRs "distinct", we applied two further criteria. First, if a PIR interacted with another alternative promoter at a lenient CHiCAGO score ≥3, we defined that PIR as shared. Second, if the adjacent fragment to the PIR in question interacted with another alternative promoter at a CHiCAGO score ≥3, we also defined that PIR as shared. We note that, under our classification rules, the PIRs of genes with only two alternative promoters included in the analysis can only be classified as "fully shared" or "distinct". Therefore, the "partially shared" PIR category was only applicable to the subset of genes with more than two baited alternative promoters.

**Epigenomic data pre-processing**

For epigenetic data analysis in ILC3s, the SRA accession list was downloaded from the GEO accession GSE77299. The SRA files were converted to FASTQ files and sequencing adapters were trimmed from reads using *trim galore* (https://github.com/FelixKrueger/TrimGalore). The reads were filtered by PHRED score ≥30 and examined for proper pairing with a mate (when paired-end). The sequencing quality and duplication level were checked using FastQC (https://www.bioinformatics.babraham.ac.uk/projects/fastqc/). Sequences were mapped to the hg38 reference genome using STAR with modifications for aligning ChIP-seq and ATAC-seq reads. Samtools[51] was used to select reads with a MAPQ score of 255, which is the flag for uniquely mapping reads from STAR[52]. ATAC-seq reads were filtered, retaining properly paired and oriented reads using the samflag=3. PCR duplicates were removed using samtools. We then removed reads that fell within blacklisted regions using Bedtools[53] intersect. The final filtered BAM file was then converted to a BED file using Bedtools bamtobed. This conversion breaks read-pairing and ensures each read contributes to peak identification with MACS2[54]. The ATAC-seq reads in BED format were shifted by +4 bp on the (+) strand, and -5 bp on the (-) strand to account for the Tn5 transposase cut site. Peaks were called using MACS2 using three biological replicates per sample as the treatment group with an input ChIP-seq control sample. The replicate correlation between the ATAC-seq samples was poor, with a <10% overlap between biological replicates. This result was consistent with the high level of duplication and low peak count (8,852) in the worst sample (SRR3129112). Thus, our ATAC-seq results were limited to the sample with the best quality metrics (SRR3129113). In total, we detected 34,077 H3K27ac peaks and 72,825 ATAC-seq peaks. For epigenetic data analysis in CD4+ T cells, we used BLUEPRINT epigenome datasets from male donors C002Q1, S008H1, and S007G7.

**PIR enrichment for epigenomic features**

For each gene, sets of adjacent PIRs for each gene (detected at the fragment or 5 kb resolution or the merged PIR sets for each gene) were collapsed together to obtain

"collapsed PIRs" (cPIRs). Trans-chromosomal PIRs were removed. The observed proportion of cPIRs overlapping epigenomic features of interest (ATAC-seq, H3K27ac or H3K4me3, respectively) was computed using the *foverlaps* function from the *data.table* package in R. To obtain the expectation for this proportion, we repeated this analysis for random cPIRs that were generated by "transplanting" each set of all cPIRs for each gene to randomly selected genes in a manner preserving the size and spatial localisation of the cPIRs with respect to each other and the respective baited promoter fragment. This "transplantation" was repeated 100 times for all genes (baited promoter fragments), and the mean proportion of random cPIRs overlapping epigenomic features of interest (over 100 permutations), as well as the standard deviation of this quantity, were compared with the proportion of overlap for the observed cPIRs. Compared with the PIR enrichment estimation algorithm implemented in CHiCAGO (*peakEnrichment4Features*), this permutation procedure preserves not only each PIR's distance from bait, but also the spatial relationships between multiple PIRs of the same gene.

**Assessing the biological function of CD-prioritised genes**

The Gene2Func tool in FUMA (v1.5.2) was run using all multiCOGS genes with a score ≥0.5, Ensembl version 102, and GTex v8. As a background, we used all genes with assigned multiCOGS scores in ILC3s, of which 17,984 had a recognised Ensembl Gene ID in FUMA. Multiple testing correction was done via the Benjamini-Hochberg method (FDR) with an adjusted p-value cutoff of 0.05 and a minimum of 2 genes in a set. The MsigDB[3] version was v7.0. We additionally checked for enrichment of multiCOGS genes in The Inflammatory Bowel Disease Transcriptome and Metatranscriptome Meta-Analysis (IBD TaMMA) Framework[7]. We filtered the 496 datasets of differentially expressed (DE) genes (adjusted p-value < 0.05 and absolute log2 fold change ≥2) that were compared across the same tissues and selected only sets with a maximum of 2,000 DE genes, to avoid mis-estimation of the normalised enrichment score, resulting in 24 datasets. Then we ran the *enricher* function in the R package clusterProfiler[55] (version 4.2.2) for all multiCOGS genes with a score ≥0.5.

**Classic COGS**

To run classic COGS[2,25], we adapted the code from the R package rCOGS (https://github.com/ollyburren/rCOGS) to use the *data.table* framework instead of *GenomicRanges* for optimised speed and to enable both the classic COGS and multiCOGS analyses (see Code availability). We used linkage disequilibrium blocks calculated for GRCh38 from https://github.com/jmacdon/LDblocks_GRCh38[56] and minor allele frequencies from the 1000 Genomes Project for European individuals.

**Sources of prior mechanistic evidence for CD genes**

Datasets used to compare the COGS prioritised genes with previously functionally validated genes were: OpenTargets[37] (L2G gene prioritisation score > 0.5 for five CD studies[57–61]), the IBDDB database of functionally validated targets[62], a functional screen of IBD genes[63], experimentally validated IBD and CD genes from DisGeNET[64] that had evidence "AlteredExpression", "Biomarker", "Posttranslationalmodification", or "Therapeutic" or CD-containing exonic variants in a recent IBD exome study[65].

### Stratified LD score regression (LDSC) analysis

We used PIR coordinates and the per-chromosome .bim files for Phase 3 1000Genome EUR individuals (downloaded from https://alkesgroup.broadinstitute.org/LDSCORE/GRCh38) to create LDSC annotations using *make_annot.py* from the LDSC package[66]. CD summary stats from de Lange et al.[61] were downloaded from the GWAS catalog (accession GCST004132) and reformatted using MungeSumstats[67] and dbSNP 155 annotations. LDSC statistics were estimated using ldsc.py in LD windows +/- 1cM around HapMap 3 SNPs.

### LOLA enrichment analysis

We performed LOLA v1.18[68] enrichment analysis to assess whether active and/or open regulatory elements of multiCOGS-prioritised genes were enriched for specific transcription factor binding sites and chromatin features compared to all genes tested by multiCOGS. We focused on PIRs with overlapping ATAC-seq or H3K27ac ChIP-seq peaks ("active PIRs"). The background universe comprised all active PIRs from the same datasets for all tested genes. Regions were converted to GRanges objects using the GenomicRanges package, and enrichment was tested using the LOLA core pipeline with the LOLA Core RegionDB, using default parameters. Significant enrichments were defined as those with q-value < 0.05.

### Supplementary references


1. Nasser, J. *et al.* Genome-wide enhancer maps link risk variants to disease genes. *Nature* **593**, 238–243 (2021).
2. Javierre, B. M. *et al.* Lineage-Specific Genome Architecture Links Enhancers and Non-coding Disease Variants to Target Gene Promoters. *Cell* **167**, 1369–1384.e19 (2016).
3. Liberzon, A. *et al.* The Molecular Signatures Database (MSigDB) hallmark gene set collection. *Cell Syst* **1**, 417–425 (2015).
4. He, P. *et al.* A human fetal lung cell atlas uncovers proximal-distal gradients of differentiation and key regulators of epithelial fates. *Cell* **185**, 4841–4860.e25 (2022).
5. Victor, A. R. *et al.* IL-18 drives ILC3 proliferation and promotes IL-22 production via NF-κB. *J. Immunol.* **199**, 2333–2342 (2017).
6. Agrawal, A. *et al.* WikiPathways 2024: next generation pathway database. *Nucleic Acids Res* **52**, D679–D689 (2024).
7. Massimino, L. *et al.* The Inflammatory Bowel Disease Transcriptome and Metatranscriptome Meta-Analysis (IBD TaMMA) framework. *Nature Computational Science* vol. 1 511–515 Preprint at https://doi.org/10.1038/s43588-021-00114-y (2021).
8. Honap, S. *et al.* JAK inhibitors for inflammatory bowel disease: recent advances. *Frontline Gastroenterol.* **15**, 59–69 (2024).
9. Wakefield, J. A Bayesian measure of the probability of false discovery in genetic epidemiology studies. *Am J Hum Genet* **81**, 208–227 (2007).
10. Zou, Y., Carbonetto, P., Wang, G. & Stephens, M. Fine-mapping from summary data with the 'Sum of Single Effects' model. *PLoS Genet.* **18**, e1010299 (2022).
11. Võsa, U. *et al.* Large-scale cis- and trans-eQTL analyses identify thousands of genetic loci and polygenic scores that regulate blood gene expression. *Nature Genetics* **53**,

1300–1310 (2021).
12. Sloan, C. A. *et al.* ENCODE data at the ENCODE portal. *Nucleic Acids Res.* **44**, D726–32 (2016).
13. Guenther, M. G., Levine, S. S., Boyer, L. A., Jaenisch, R. & Young, R. A. A chromatin landmark and transcription initiation at most promoters in human cells. *Cell* **130**, 77–88 (2007).
14. Chantalat, S. *et al.* Histone H3 trimethylation at lysine 36 is associated with constitutive and facultative heterochromatin. *Genome Res.* **21**, 1426–1437 (2011).
15. Carvalho-Silva, D. *et al.* Open Targets Platform: new developments and updates two years on. *Nucleic Acids Res.* **47**, D1056–D1065 (2018).
16. Giambartolomei, C. *et al.* Bayesian test for colocalisation between pairs of genetic association studies using summary statistics. *PLoS Genet.* **10**, e1004383 (2014).
17. Lepik, K. *et al.* C-reactive protein upregulates the whole blood expression of CD59 - an integrative analysis. *PLoS Comput. Biol.* **13**, e1005766 (2017).
18. GTEx Consortium. The Genotype-Tissue Expression (GTEx) project. *Nat. Genet.* **45**, 580–585 (2013).
19. Quach, H. *et al.* Genetic Adaptation and Neandertal Admixture Shaped the Immune System of Human Populations. *Cell* **167**, 643–656.e17 (2016).
20. Chen, L. *et al.* Genetic Drivers of Epigenetic and Transcriptional Variation in Human Immune Cells. *Cell* **167**, 1398–1414.e24 (2016).
21. Michieletto, M. F. *et al.* Multiscale 3D genome organization underlies ILC2 ontogenesis and allergic airway inflammation. *Nat. Immunol.* 1–13 (2022).
22. Ho, J. S. Y. *et al.* TOP1 inhibition therapy protects against SARS-CoV-2-induced lethal inflammation. *Cell* **184**, 2618–2632.e17 (2021).
23. Fulco, C. P. *et al.* Activity-by-contact model of enhancer-promoter regulation from thousands of CRISPR perturbations. *Nat. Genet.* **51**, 1664–1669 (2019).
24. Koues, O. I. *et al.* Distinct Gene Regulatory Pathways for Human Innate versus Adaptive Lymphoid Cells. *Cell* **165**, 1134–1146 (2016).
25. Burren, O. S. *et al.* Chromosome contacts in activated T cells identify autoimmune disease candidate genes. *Genome Biol.* **18**, 165 (2017).
26. Ray-Jones, H. & Spivakov, M. Transcriptional enhancers and their communication with gene promoters. *Cell. Mol. Life Sci.* **78**, 6453–6485 (2021).
27. Abell, N. S. *et al.* Multiple causal variants underlie genetic associations in humans. *Science* **375**, 1247–1254 (2022).
28. Castel, S. E. *et al.* Modified penetrance of coding variants by cis-regulatory variation contributes to disease risk. *Nat. Genet.* **50**, 1327–1334 (2018).
29. Maurano, M. T. *et al.* Systematic localization of common disease-associated variation in regulatory DNA. *Science* **337**, 1190–1195 (2012).
30. Dryden, N. H. *et al.* Unbiased analysis of potential targets of breast cancer susceptibility loci by Capture Hi-C. *Genome Res* **24**, 1854–1868 (2014).
31. He, H. *et al.* Multiple functional variants in long-range enhancer elements contribute to the risk of SNP rs965513 in thyroid cancer. *Proc Natl Acad Sci U S A* **112**, 6128–6133 (2015).
32. Lawrenson, K. *et al.* Cis-eQTL analysis and functional validation of candidate susceptibility genes for high-grade serous ovarian cancer. *Nat Commun* **6**, 8234 (2015).
33. Costanzo, M. C. *et al.* Realizing the promise of genome-wide association studies for effector gene prediction. *Nat. Genet.* **57**, 1578–1587 (2025).
34. Watanabe, K., Taskesen, E., van Bochoven, A. & Posthuma, D. Functional mapping and

annotation of genetic associations with FUMA. *Nat. Commun.* **8**, 1826 (2017).
35. Mountjoy, E. *et al.* An open approach to systematically prioritize causal variants and genes at all published human GWAS trait-associated loci. *Nat. Genet.* **53**, 1527–1533 (2021).
36. McLaren, W. *et al.* The Ensembl Variant Effect Predictor. *Genome Biol* **17**, 122 (2016).
37. Ochoa, D. *et al.* Open Targets Platform: supporting systematic drug-target identification and prioritisation. *Nucleic Acids Res.* **49**, D1302–D1310 (2021).
38. Sey, N. Y. A. *et al.* A computational tool (H-MAGMA) for improved prediction of brain-disorder risk genes by incorporating brain chromatin interaction profiles. *Nat. Neurosci.* **23**, 583–593 (2020).
39. Barral, A. *et al.* SETDB1/NSD-dependent H3K9me3/H3K36me3 dual heterochromatin maintains gene expression profiles by bookmarking poised enhancers. *Mol. Cell* **82**, 816–832.e12 (2022).
40. Bonder, M. J. *et al.* Identification of rare and common regulatory variants in pluripotent cells using population-scale transcriptomics. *Nat. Genet.* **53**, 313–321 (2021).
41. Cui, Y. *et al.* 3'aQTL-atlas: an atlas of 3'UTR alternative polyadenylation quantitative trait loci across human normal tissues. *Nucleic Acids Res.* **50**, D39–D45 (2022).
42. Uriostegui-Arcos, M., Mick, S. T., Shi, Z., Rahman, R. & Fiszbein, A. Splicing-dependent transcriptional activation. *bioRxiv* 2022.09.16.508316 (2022) doi:10.1101/2022.09.16.508316.
43. Kerimov, N. *et al.* A compendium of uniformly processed human gene expression and splicing quantitative trait loci. *Nat. Genet.* **53**, 1290–1299 (2021).
44. Borén, J., Packard, C. J. & Binder, C. J. Apolipoprotein B-containing lipoproteins in atherogenesis. *Nat Rev Cardiol* **22**, 399–413 (2025).
45. Lockstone, H. E. Exon array data analysis using Affymetrix power tools and R statistical software. *Brief. Bioinform.* **12**, 634–644 (2011).
46. Akgol Oksuz, B. *et al.* Systematic evaluation of chromosome conformation capture assays. *Nat. Methods* **18**, 1046–1055 (2021).
47. Su, C., Pahl, M. C., Grant, S. F. A. & Wells, A. D. Restriction enzyme selection dictates detection range sensitivity in chromatin conformation capture-based variant-to-gene mapping approaches. *Hum. Genet.* **140**, 1441–1448 (2021).
48. Freire-Pritchett, P. *et al.* Detecting chromosomal interactions in Capture Hi-C data with CHiCAGO and companion tools. *Nat. Protoc.* **16**, 4144–4176 (2021).
49. Cairns, J. *et al.* CHiCAGO: robust detection of DNA looping interactions in Capture Hi-C data. *Genome Biol.* **17**, 127 (2016).
50. McInnes, L., Healy, J. & Melville, J. UMAP: Uniform Manifold Approximation and Projection for Dimension Reduction. (2018) doi:10.48550/arXiv.1802.03426.
51. Li, H. *et al.* The Sequence Alignment/Map format and SAMtools. *Bioinformatics* **25**, 2078–2079 (2009).
52. Dobin, A. *et al.* STAR: ultrafast universal RNA-seq aligner. *Bioinformatics* **29**, 15–21 (2013).
53. Quinlan, A. R. & Hall, I. M. BEDTools: a flexible suite of utilities for comparing genomic features. *Bioinformatics* **26**, 841–842 (2010).
54. Gaspar, J. M. Improved peak-calling with MACS2. *bioRxiv* (2018) doi:10.1101/496521.
55. Yu, G., Wang, L.-G., Han, Y. & He, Q.-Y. clusterProfiler: an R package for comparing biological themes among gene clusters. *OMICS* **16**, 284–287 (2012).
56. MacDonald, J. W., Harrison, T., Bammler, T. K., Mancuso, N. & Lindström, S. An updated map of GRCh38 linkage disequilibrium blocks based on European ancestry

data. *Biorxiv* (2022) doi:10.1101/2022.03.04.483057.
57. Barrett, J. C. *et al.* Genome-wide association defines more than 30 distinct susceptibility loci for Crohn's disease. *Nat. Genet.* **40**, 955–962 (2008).
58. Franke, A. *et al.* Genome-wide meta-analysis increases to 71 the number of confirmed Crohn's disease susceptibility loci. *Nat. Genet.* **42**, 1118–1125 (2010).
59. Liu, J. Z. *et al.* Association analyses identify 38 susceptibility loci for inflammatory bowel disease and highlight shared genetic risk across populations. *Nat. Genet.* **47**, 979–986 (2015).
60. Ellinghaus, D. *et al.* Analysis of five chronic inflammatory diseases identifies 27 new associations and highlights disease-specific patterns at shared loci. *Nat. Genet.* **48**, 510–518 (2016).
61. de Lange, K. M. *et al.* Genome-wide association study implicates immune activation of multiple integrin genes in inflammatory bowel disease. *Nat. Genet.* **49**, 256–261 (2017).
62. Khan, F., Radovanovic, A., Gojobori, T. & Kaur, M. IBDDB: a manually curated and text-mining-enhanced database of genes involved in inflammatory bowel disease. *Database* **2021**, baab022 (2021).
63. Ntunzwenimana, J. C. *et al.* Functional screen of inflammatory bowel disease genes reveals key epithelial functions. *Genome Med.* **13**, 181 (2021).
64. Piñero, J. *et al.* DisGeNET: a comprehensive platform integrating information on human disease-associated genes and variants. *Nucleic Acids Res.* **45**, D833–D839 (2016).
65. Sazonovs, A. *et al.* Large-scale sequencing identifies multiple genes and rare variants associated with Crohn's disease susceptibility. *Nat. Genet.* 1–9 (2022).
66. Finucane, H. K. *et al.* Partitioning heritability by functional annotation using genome-wide association summary statistics. *Nat. Genet.* **47**, 1228–1235 (2015).
67. Murphy, A. E., Schilder, B. M. & Skene, N. G. MungeSumstats: a Bioconductor package for the standardization and quality control of many GWAS summary statistics. *Bioinformatics* **37**, 4593–4596 (2021).
68. Sheffield, N. C. & Bock, C. LOLA: enrichment analysis for genomic region sets and regulatory elements in R and Bioconductor. *Bioinformatics* **32**, 587–589 (2016).

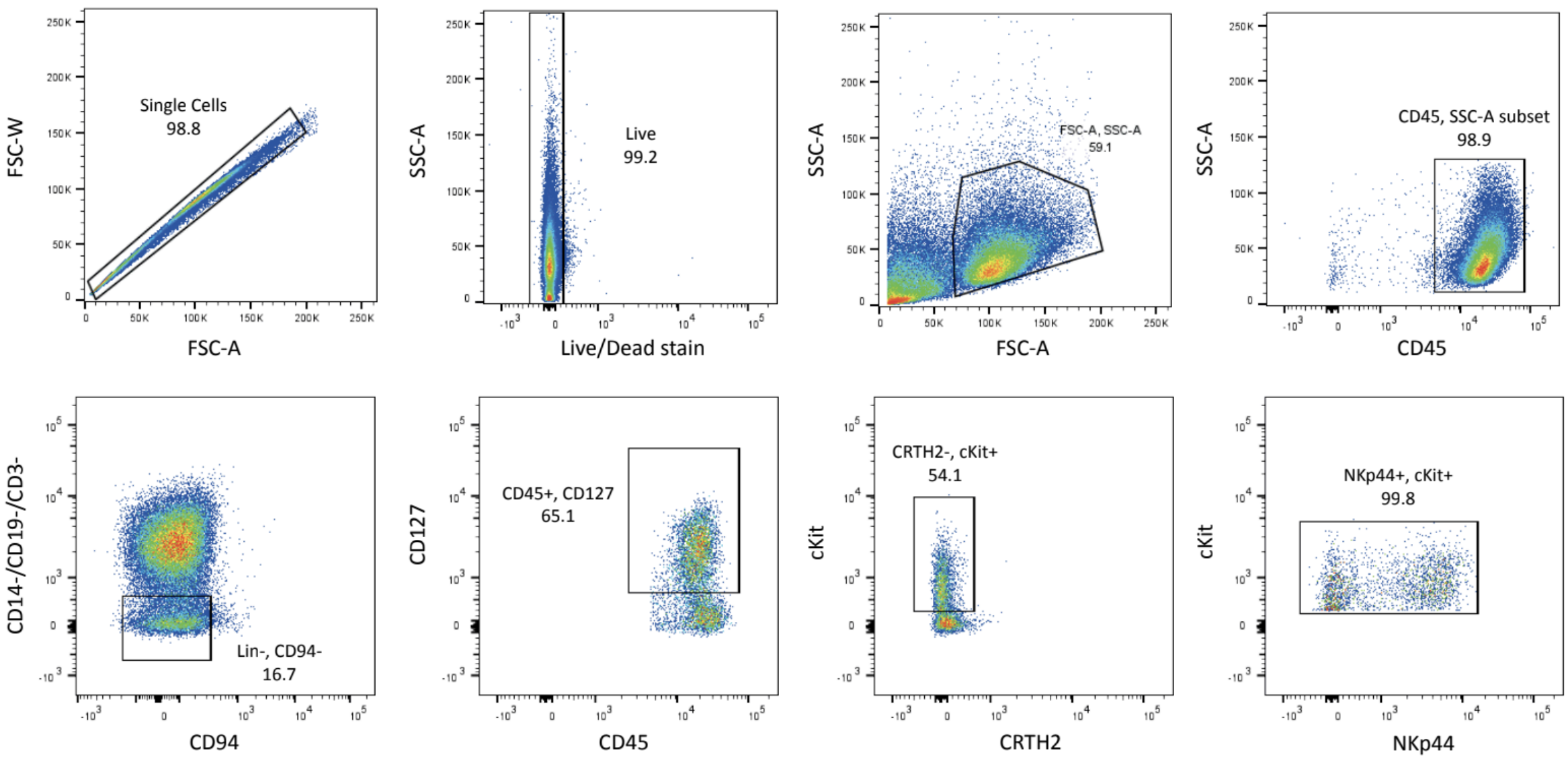


**Supplementary Figure 1. Flow cytometry gating strategy for isolation of human ILC3s from tonsils.**

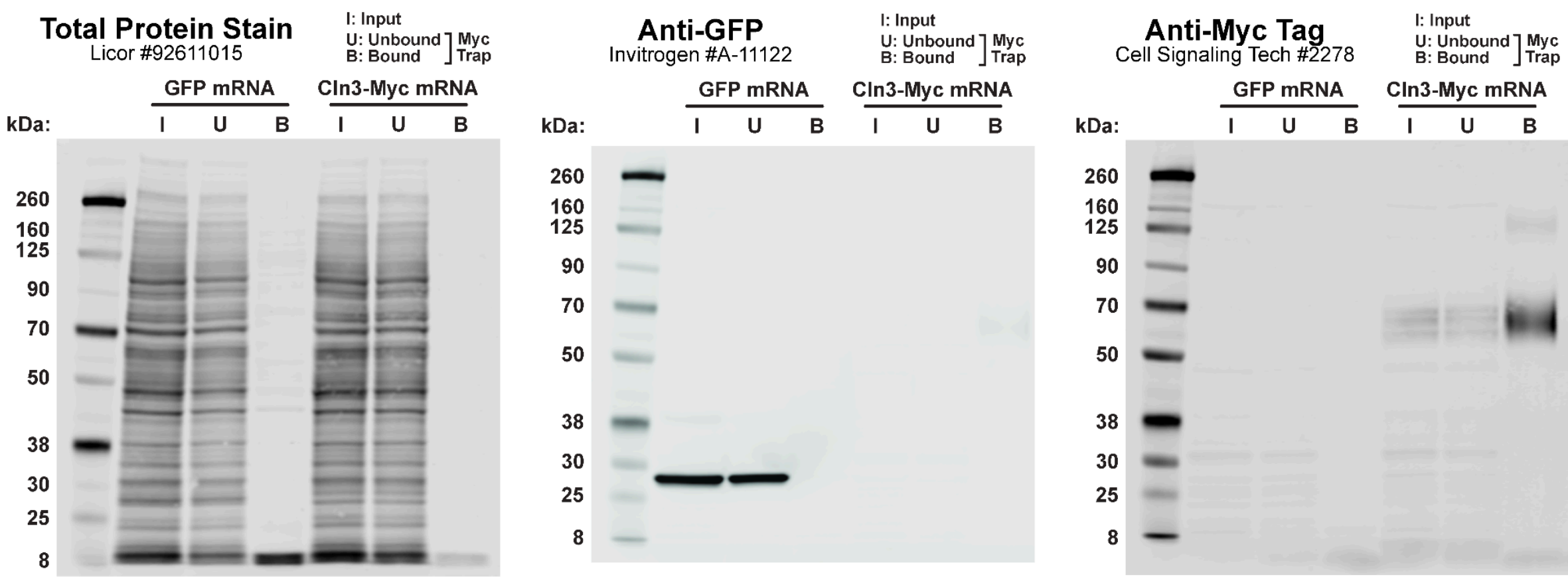


**Supplementary Figure 2. Uncropped gel image, supporting Extended Data Figure 8.**
