# Supplementary material for: High-resolution promoter interaction analysis implicates genes involved in activation of type 3 innate lymphoid cells in immune disease risk
Source: Nat Genet. 2026 Aug 4;58(8):1953–66. doi: 10.1038/s41588-026-02681-0 (PMC13447110; doi:10.1038/s41588-026-02681-0)
Supplement: Supplementary file 2 [file 41588_2026_2681_MOESM2_ESM.pdf]

nature portfolio

Corresponding author(s): Valeriya Malysheva
Stephen Waggoner
Mikhail Spivakov

Last updated by author(s): 10th June 2026

Reporting Summary

Nature Portfolio wishes to improve the reproducibility of the work that we publish. This form provides structure for consistency and transparency in reporting. For further information on Nature Portfolio policies, see our Editorial Policies and the Editorial Policy Checklist.

Statistics

For all statistical analyses, confirm that the following items are present in the figure legend, table legend, main text, or Methods section.

n/a	Confirmed	
☐	☒	The exact sample size (*n*) for each experimental group/condition, given as a discrete number and unit of measurement
☐	☒	A statement on whether measurements were taken from distinct samples or whether the same sample was measured repeatedly
☐	☒	The statistical test(s) used AND whether they are one- or two-sided<br>*Only common tests should be described solely by name; describe more complex techniques in the Methods section.*
☐	☒	A description of all covariates tested
☐	☒	A description of any assumptions or corrections, such as tests of normality and adjustment for multiple comparisons
☐	☒	A full description of the statistical parameters including central tendency (e.g. means) or other basic estimates (e.g. regression coefficient) AND variation (e.g. standard deviation) or associated estimates of uncertainty (e.g. confidence intervals)
☐	☒	For null hypothesis testing, the test statistic (e.g. *F*, *t*, *r*) with confidence intervals, effect sizes, degrees of freedom and *P* value noted<br>*Give P values as exact values whenever suitable.*
☒	☐	For Bayesian analysis, information on the choice of priors and Markov chain Monte Carlo settings
☒	☐	For hierarchical and complex designs, identification of the appropriate level for tests and full reporting of outcomes
☒	☐	Estimates of effect sizes (e.g. Cohen's *d*, Pearson's *r*), indicating how they were calculated

Our web collection on statistics for biologists contains articles on many of the points above.

Software and code

Policy information about availability of computer code

Data collection	No software was used for data collection
Data analysis	PCHi-C data was pre-processed and analysed using Bowtie2, HiCUP and CHiCAGO.<br>ChIP-seq data was pre-processed using Trim galore, Fastqc, STAR, Samtools and Bedtools and peaks were called using MACS2.<br>Microarray CEL files were analysed using AltAnalyze.<br>Human RNA-seq was processed using Salmon and Tx import.<br>Mouse RNA-seq was analysed using kallisto (v0.48.0), tximport (v1.30.0) and DESeq2 (v1.38.0).<br>Enrichment of TFs in active and/or open regulatory elements of multiCOGS-prioritised genes was deteced using LOLA v1.18.<br>Enrichment of trait-associated variants within open + active PIRs in ILC3s and CD4+ T cells was detected using RELI v0.1.1a.<br>Liftover of genomic regions was performed using UCSC LiftOver v377.<br>The mutiCOGS algorithm is based on the R package rCOGS, leveraging the R package data.table. To identify coding SNPs in the workflow, we used VEP version 99.2. The multivariate fine mapping integrated into multiCOGS was performed using the susieR package.<br>Pathway analysis in the multiCOGS vs CRISPRi screen genes was performed using MAGeCK v0.5.9.5.<br>The scripts used to generate the data in this study are freely available on Github. All repositories containing code used in this study are listed under the topic "malysheva et al", available at: https://github.com/topics/malysheva-et-al

For manuscripts utilizing custom algorithms or software that are central to the research but not yet described in published literature, software must be made available to editors and reviewers. We strongly encourage code deposition in a community repository (e.g. GitHub). See the Nature Portfolio guidelines for submitting code & software for further information.

Data

Policy information about availability of data

All manuscripts must include a data availability statement. This statement should provide the following information, where applicable:

- Accession codes, unique identifiers, or web links for publicly available datasets
- A description of any restrictions on data availability
- For clinical datasets or third party data, please ensure that the statement adheres to our policy

Raw Promoter Capture Hi-C data generated in this study are deposited in the Gene Expression Omnibus (GEO) under the accession number GSE216267 (ILC3s) and in the European Genome-Phenome Archive (EGA) under managed access, accession number EGAS50000001316 (owing to conditions of donor consent). Raw RNA-seq reads and counts for the CRISPRi/a experiments at CLN3 in MNK3 cells are deposited in GEO under the accession number GSE313942. Supplementary Data files, including significant CHiCAGO interactions at fragment-level and 5kb resolution in ILC3 and CD4+ T cells, ABCC pairs in both cell types and DESeq2 objects for the CLN3 CRISPRi/a experiments, were deposited to Open Science Framework (https://osf.io/aq9fb).

Human research participants

Policy information about studies involving human research participants and Sex and Gender in Research.

Reporting on sex and gender	Two male and one female child participated in our study for ILC3s and two adult male donors for CD4+ T cells
Population characteristics	ILC3s:<br>Age: 6, Non-Hispanic European American<br>Age:5, Non-Hispanic African American<br>Age:6, Non-Hispanic European American<br><br>CD4+ T cells:<br>Two adult male donors of European ancestry (age not disclosed due to consent conditions)
Recruitment	ILC3: Three children requiring tonsillectomy were recruited to a prospective study at a tertiary academic care center through the division of Pediatric Otolaryngology-Head and Neck surgery at Cincinnati Children's Hospital Medical Center. Criteria for enrollment in the study included a history of sleep-disordered breathing or recurrent or chronic tonsillitis requiring removal of the tonsillar tissue. Subjects were excluded from the study if the tonsillar tissue was acutely infected or if anatomic abnormalities were present requiring a more detailed pathologic evaluation post the surgical procedure.<br><br>CD4+ T cells: Donors were recruited through NIHR Cambridge BioResource (UK) to donate blood for research purposes at an NHS Blood and Transplant facility at the University of Cambridge.<br><br>We have not identified any bias in the recruitment of participants.
Ethics oversight	ILC3: Consent was obtained from parents in the perioperative suite on the day of the procedure with approval of the institutional review board (IRB) “Tissues for the study of functional genomics and immunity” (no. 2018-3069) at the Cincinnati Children's Hospital Medical Center.<br>CD4+ T cells: The samples were obtained after written informed consent under studies “A Blueprint of Blood Cells,” REC reference 12/EE/0040, and “Genes and mechanisms in type 1 diabetes in the Cambridge BioResource,” REC reference 05/Q0106/20; both NRES Committee East of England – Cambridgeshire and Hertfordshire.<br>Both: Samples were labelled with a de-identified barcode and transferred to the research team for further processing.

Note that full information on the approval of the study protocol must also be provided in the manuscript.

Field-specific reporting

Please select the one below that is the best fit for your research. If you are not sure, read the appropriate sections before making your selection.

☒ Life sciences ☐ Behavioural & social sciences ☐ Ecological, evolutionary & environmental sciences

For a reference copy of the document with all sections, see nature.com/documents/nr-reporting-summary-flat.pdf

Life sciences study design

All studies must disclose on these points even when the disclosure is negative.

Sample size	To study chromatin organistaion we generated Promoter Capture Hi-C data for two (CD4+ T cells) or three (ILC3 cells) biological replicates. For the CRISPR activation/interference experiments, we included three biological replicates per condition.

For the CLN3 overexpression experiments, we performed two independent experiments; the first with three biological replicates and the second with 10 biological replicates.

Data exclusions

No data was excluded from the analysis.

Replication

The number of replicates was determined based on material availability and common practice for each experiment type. For Promoter Capture Hi-C, the use of two biological replicates is standard in the field and sufficient for detecting significant interactions using the CHiCAGO methodology (Freire-Pritchett/Ray-Jones et al., Nature Protocols 2021). For the other experiments, we did not pre-determine sample sizes based on statistical methodologies. We have not reported any findings that failed replication.

Randomization

Randomization is not relevant for this study, due to the focus on a specific cell type in control samples.

Blinding

Blinding is not relevant for this study, due to the focus on a specific cell type in control samples.

Reporting for specific materials, systems and methods

We require information from authors about some types of materials, experimental systems and methods used in many studies. Here, indicate whether each material, system or method listed is relevant to your study. If you are not sure if a list item applies to your research, read the appropriate section before selecting a response.

Materials & experimental systems

n/a	Involved in the study	
☐	☒	Antibodies
☐	☒	Eukaryotic cell lines
☒	☐	Palaeontology and archaeology
☒	☐	Animals and other organisms
☒	☐	Clinical data
☒	☐	Dual use research of concern

Methods

n/a	Involved in the study	
☐	☒	ChIP-seq
☐	☒	Flow cytometry
☒	☐	MRI-based neuroimaging

Antibodies

Antibodies used

Human
CD19 (HIB19) BV421 1:50 Biolegend #302234
CD14 (63D3) BV421 1:50 Biolegend #367144
CD3 (OKT3) BV421 1:50 Biolegend #317344
CD45 (HI30) AF488 1:200 Biolegend #304017
CD94 (DX22) PerCP-Cy5.5 1:50 Biolegend #305514
CD127 (IL-7Rα) (A019D5) PE-Cy7 1:50 Biolegend #351320
cKit (CD117) (104D2) BV510 1:50 Biolegend #313220
NKp44 (P44-8) AF647 1:50 Biolegend #325112
CRTH2 (301109) PE 1:50 R&D Systems #FAB33381P
Live/dead fixable near-IR Dead cell stain kit 1:1000 Invitrogen #L34993
Human TruStain FcX™ 1:50 Biolegend #422302

Mouse
CCR6 (29-2L17) BV421 1:100 Biolegend #129818
CD3 (17A2) BUV395 1:200 BD Biosciences #740268
CD19 (1D3) BUV395 1:200 BD Biosciences #563557
TCR β Chain (H57-597) BUV395 1:200 BD Biosciences #742485
NK1.1 (PK136) BV711 1:50 Biolegend #108745
NKp46 (CD335) (29A1.4) 1:50 Biolegend #137619
KLRG1 (2F1) FITC 1:100 Biolegend #138410
CD127 (IL-7Rα) (A7R34) PE/Cy7 1:100 BioLegend #135014
CD69 (Y245) BUV737 1:100 BD Biosciences #612793
IL-17A (TC1118H10.1) PE 1:50 BioLegend #506904
CD16/CD32 (2.4G2) 1:200 Tonbo #70-0161-M001
ELISA DuoSet kits for mouse IL-17, IL-22, and GM-CSF (R&D Systems).
Live/dead fixable near-IR Dead cell stain kit 1:1000 Invitrogen #L34993

Validation

Antibody validation is provided on the supplier website(s). All antibodies were validated with proper FMO controls using PBMCs or leukocytes derived from human tonsils.
Supplier validation from Biolegend can be found at: https://www.biolegend.com/en-us/quality/quality-control.
R&D: https://www.rndsystems.com/products/antibodies.
BD Biosciences: https://www.bdbiosciences.com/en-us/products/reagents/flow-cytometry-reagents/research-reagents/quality-and-reproducibility.

Eukaryotic cell lines

Policy information about cell lines and Sex and Gender in Research

Cell line source(s)	MNK-3a and MNK-3i cells were generated in the Oltz lab based on the MNK-3 male mouse cell line (Sigma-Aldrich, SCC628).
Authentication	The cells were not authenticated.
Mycoplasma contamination	The cell lines were mycoplasma-negative at the time of analysis.
Commonly misidentified lines (See ICLAC register)	None

ChIP-seq

Data deposition

☒ Confirm that both raw and final processed data have been deposited in a public database such as GEO.

☒ Confirm that you have deposited or provided access to graph files (e.g. BED files) for the called peaks.

Data access links *May remain private before publication.*	No raw ChIP-seq data was generated in this study. Publicly available ChIP-seq data was downloaded from GEO, accession GSE77299. Processed ChIP-seq files are available on: https://github.com/tacazares/spivakov_pchic_ILC_CD4,
Files in database submission	No raw ChIP-seq generated in this study
Genome browser session (e.g. UCSC)	No raw ChIP-seq generated in this study

Methodology

Replicates	Three biological replicates for each assay
Sequencing depth	as described in the original study GSE77299
Antibodies	H3K27ac, H3K4me3
Peak calling parameters	Peaks were called using MACS2 using three biological replicates per sample as the treatment group with an input ChIP-seq control sample. Deafult parameters.
Data quality	The sequencing reads were filtered by PHRED score >=30 and examined for proper pairing with a mate (when paired-end). The sequencing quality and duplication level were checked using Fastqc. Sequences were mapped to the hg38 reference genome using STAR with modification. Samtools was used to select reads with a MAPQ score of 255 which is the flag for unique mapping reads from STAR. PCR duplicates were removed using samtools. We then removed reads that fell within blacklisted regions using Bedtools intersect. The final filtered BAM file was then converted to a bed file using Bedtools bamtobed.
Software	MACS2

Flow Cytometry

Plots

Confirm that:

☒ The axis labels state the marker and fluorochrome used (e.g. CD4-FITC).

☒ The axis scales are clearly visible. Include numbers along axes only for bottom left plot of group (a 'group' is an analysis of identical markers).

☒ All plots are contour plots with outliers or pseudocolor plots.

☒ A numerical value for number of cells or percentage (with statistics) is provided.

Methodology

Sample preparation	ILC3 isolation: Human tonsil tissue was processed by mincing with scissors followed by transfer of up to 4g of tissue to a gentleMACS C tube (Miltenyi Biotec) containing 8 mL of phosphate-buffered saline (PBS) with 0.5 mg/mL collagenase D and 3000 U/mL DNaseI, then dissociated on a GentleMACS Octo Dissociator (Miltenyi Biotec) using "program C (Spleen program 2 followed by spleen program 3)." Tissue homogenates were incubated in a 37°C water bath for 15 minutes, then dissociated again using "program C" and transferred through a 100 µm cell strainer into 20mL RPMI containing 10% human AB serum (Sigma Aldrich). Next, cell suspension was overlaid on 10mL of Ficoll-Paque PLUS (GE Healthcare) and subjected to density-gradient separation via centrifugation for 20 min at 1800rpm, 20°C, slow acceleration, no brake. Leukocytes were collected from the interphase layer and then washed with 50mL of PBS for 6 minutes at 1600rpm for 7 minutes, 20°C.

Single cell suspensions of tonsil mononuclear cells were subjected to positive selection with anti-human-CD3, anti-human-CD19 and anti-human-CD14 (Miltenyi Biotec) and transferred through LD columns (Miltenyi Biotec) according to the manufacturer guidelines. The depleted cell suspension flowthrough was collected into 15mL conical and then centrifuged for 5 minutes at 1200rpm, 20°C. Subsequently, cells were labeled with LIVE/DEAD™ Fixable Near-IR dead cell stain kit (Invitrogen). Next, cells were labeled with sorting antibody cocktail which contained negative linage (Lin-) CD19 Brilliant Violet (BV)421 (HIB19), CD14-BV421 (63D3) and CD3-BV421 (OKT3), and the following antibodies: CD45- FITC, (HI30), CD94-PerCP-Cy5.5 (DX22), CD127-PE-Cy7 (A019D5), cKit-BV510 (104D2) and NKp44-Alexa Fluor (AF)647 (P44-8) all purchased from Biolegend (San Diego, CA), CRTH2-PE (301109, R&D). ILC3 cells were sorted based on the expression of CD45+Lin-CD127+CD94-CRTH2-cKit+NKp44+, similarly to Bar-Ephraim et al.

Instrument

Human ILC3s: Cell sorting was performed using a FACSAria II sorter (BD Biosciences, Mountain View, CA, USA).

Software

All data were analyzed using FlowJo (Tree Star).

Cell population abundance

cKit+/- NKp44+ sorted cells ranged between 0.3% to 2.9% from total amount of sorted cells. The amount of sorted cells ranged between 100,000 to 216,000 per sort.

Gating strategy

ILC3s were sorted based on the expression of CD45+Lin-CD127+CD94-CRTH2-cKit+NKp44+

☒ Tick this box to confirm that a figure exemplifying the gating strategy is provided in the Supplementary Information.
